# RNA Sequencing Reveals Specific Transcriptomic Signatures Distinguishing Effects of the [*SWI*^+^] Prion and *SWI1* Deletion in Yeast *Saccharomyces cerevisiae*

**DOI:** 10.3390/genes10030212

**Published:** 2019-03-12

**Authors:** Yury V. Malovichko, Kirill S. Antonets, Anna R. Maslova, Elena A. Andreeva, Sergey G. Inge-Vechtomov, Anton A. Nizhnikov

**Affiliations:** 1 Laboratory for Proteomics of Supra-Organismal Systems, All-Russia Research Institute for Agricultural Microbiology (ARRIAM), Podbelskogo sh., 3, Pushkin, 196608 St. Petersburg, Russia; 271296251017a@gmail.com (Y.V.M.); kirantonez@gmail.com (K.S.A.); 2 Department of Genetics and Biotechnology, St. Petersburg State University, Universitetskaya nab., 7/9, 199034 St. Petersburg, Russia; shuvalova.a.r@gmail.com (A.R.M.); l_andreeva@yahoo.com (E.A.A.); ingevechtomov@gmail.com (S.G.I.-V.); 3 Vavilov Institute of General Genetics, St. Petersburg Branch, Universitetskaya nab., 7/9, 199034 St. Petersburg, Russia

**Keywords:** prion, amyloid, yeast, *S. cerevisiae*, transcriptome, RNA-Seq, Swi1, [*SWI*^+^], SWI/SNF

## Abstract

Prions are infectious, self-perpetuating protein conformers. In mammals, pathological aggregation of the prion protein causes incurable neurodegenerative disorders, while in yeast *Saccharomyces cerevisiae*, prion formation may be neutral or even beneficial. According to the prevailing contemporary point of view, prion formation is considered to be a functional inactivation of the corresponding protein whose conformational state shifts from the functional monomeric one to the infectious aggregated one. The Swi1 protein forms the [*SWI*^+^] prion and belongs to the nucleosome remodeler complex SWI/SNF controlling the expression of a significant part of the yeast genome. In this work, we performed RNA sequencing of isogenic *S. cerevisiae* strains grown on the media containing galactose as the sole carbon source. These strains bore the [*SWI*^+^] prion or had its structural gene *SWI1* deleted. The comparative analysis showed that [*SWI*^+^] affects genome expression significantly weaker as compared to the *SWI1* deletion. Moreover, in contrast to [*SWI*^+^], the *SWI1* deletion causes the general inhibition of translation-related genes expression and chromosome I disomy. At the same time, the [*SWI*^+^] prion exhibits a specific pattern of modulation of the metabolic pathways and some biological processes and functions, as well as the expression of several genes. Thus, the [*SWI*^+^] prion only partially corresponds to the loss-of-function of *SWI1* and demonstrates several gain-of-function traits.

## 1. Introduction

In contrast to the prions of mammals [1], yeast prions determine various phenotypes stably inherited during mitosis and meiosis [2,3]. To date, about ten prions have been identified in yeast *Saccharomyces cerevisiae* [4,5]. Most of them belong to the transcriptional or translational factors or regulators [6,7,8,9,10,11], and bear glutamine and/or asparagine-rich regions participating in prion formation and propagation [3,12]. The formation of yeast prions may have harmful, neutral, or beneficial consequences for yeast cells [13,14,15]. A growing number of evidence suggests that several yeast prions may determine some adaptive traits under specific environmental conditions [10,14,15]. Nevertheless, most prions exhibit phenotypes similar to the loss-of-function mutations in the corresponding structural genes [3,16]. The molecular explanation of this phenomenon is that the prion state is typically presented by amyloid aggregates, in which the structural protein of a prion may be inactivated as opposed to its functionally active monomeric state [17]. Overall, the prions’ roles, either as regulators of adaptive traits or harmful epigenetic elements, remain ambiguous and require additional clarification.

The ATP-dependent chromatin remodeler complex SWI/SNF [18] is evolutionary conserved and found in all three domains of life [19]. In yeast, it consists of 12 subunits [20,21], most of which were initially identified in yeast screens as involved in mating type switching (*SWI*, Switching deficient) and governing carbon utilization (*SNF*, Sucrose/Non-Fermentable) [22,23]. SWI/SNF is a global regulator of expression of the yeast genome [24] and it is required for the stress-specific transcription of numerous genes [25,26]. The genes encoding SWI/SNF complex subunits are the most frequently mutated chromatin modulators in primary human tumors and act as tumor suppressors [27]. 

The Swi1 protein is a component of SWI/SNF that binds activator regions in promoters, thus it is essential for the transcription of numerous genes controlling sporulation, carbon metabolism, mating type switching, DNA replication, and repair [28,29]. Recent data have suggested that yeast SWI/SNF has a modular architecture consisting of four functional modules: (i) Arp (actin-related proteins) module, (ii) Snf2/Snf11 ATPase module, (iii) Snf5/Swi3 regulatory module, and (iv) Swi1 module [30]. Deletions of several SWI/SNF subunits incompletely disrupted SWI/SNF integrity, resulting in different genome expression patterns in the same conditions [30]. Notably, several subunits of SWI/SNF, including Swi1, may interact with transcriptional regulators individually, at least in vitro [31].

The *SWI1* deletion may be as lethal as it is viable in various yeast strains and causes some pleiotropic phenotypic manifestations, including vegetative growth defects, sporulation, and mating type switching deficiencies [32,33]. A unique feature of the *S. cerevisiae* Swi1 protein is its ability to adopt the prion state called [*SWI*^+^] [7]. The [*SWI*^+^] forms amyloid-like aggregates, and its N-terminal region rich in asparagine is essential for the maintenance and propagation of the prion [34]. Phenotypically, [*SWI*^+^] exhibits a partial loss-of-function phenotype, including sporulation and vegetative growth inhibition [7], which manifests most notably in the media containing galactose or glycerol as the sole carbon source [35]. The [*SWI*^+^] formation, similar to the *SWI1* deletion, abolishes multicellularity in several yeast strains, and this effect is related to the transcriptional repression of the *FLO* genes and may also be associated with the sequestration of several Q/N-rich transcriptional factors by the Swi1 aggregates [36]. Recently, [*SWI*^+^] has been found to modulate the translation termination efficiency, causing weak translational read-through and omnipotent suppression of the *ade1-14*_UGA_ and *trp1-289*_UAG_ nonsense alleles [35,37]. This effect only manifests in yeast strains bearing variants of the *SUP35* gene encoding the eRF3 release factor [38,39] with decreased functional activity [35,40] and was found to be associated with a decrease of the level of the *SUP45* mRNA [41,42] encoding the eRF1 release factor [38,39]. *SWI1* deletion also suppresses the phenotype of the *ade1-14*_UGA_ mutation [42], but in contrast to [*SWI*^+^] [41,42], this effect is caused by the increase of the level of the *ade1-14*_UGA_ mRNA [43]. Thus, the data obtained suggest that the effects of the [*SWI*^+^] prion and *SWI1* deletion only partially coincide and detailed transcriptome-wide analysis could facilitate a general understanding of molecular mechanisms underlying their phenotypic manifestations.

To compare the effects of Swi1 prionization and its deletional inactivation, in this work, we performed sequencing of the mRNA isolated from [*SWI*^+^], [*swi*^−^], and *swi1*Δ cells grown on medium containing galactose as the sole carbon source, where both prion and deletion cause strong growth inhibition. 

## 2. Materials and Methods 

### 2.1. Yeast Strains, Plasmids, and Cultivation Conditions

Three haploid yeast strains, 1-4-1-1-D931 [*swi*^−^], 12-1-4-1-1-D931 [*SWI*^+^], and 11-1-1-D931 *swi1*Δ [42], were used in this study. These strains are isogenic (*MAT*a *sup35*Δ:*HIS3 ade1-14 his3 leu2 lys2 ura3 trp1-289* [pL-Aβ-Sup35MC]) and differ only in the status of the Swi1 protein, which is soluble in the 1-4-1-1-D931 [*SWI*^−^], aggregated in the 12-1-4-1-1-D931 [*SWI*^+^], and absent in the 11-1-1-D931 *swi1*Δ strain. The strains contain the deletion of the *SUP35* gene encoding the eRF3 release factor [38,39] compensated for by the pL-Aβ-Sup35MC plasmid, which is also necessary to phenotypically check [*SWI*^+^] strains by the growth on the media without adenine with 100 µM CuSO_4_ (the [*SWI*^+^] yeast grow while [*swi*^−^] do not grow on such media) [35,37]. The 11-1-1-D931 *swi1*Δ strain additionally bears the deletion of the *SWI1* chromosomal copy (the region encoding the N-terminal part of the Swi1 protein, including the translation initiation codon, is deleted) [42], substituted with the *KanMX4* cassette, providing resistance of the yeast cells to geneticin (G418) aminoglycoside [44]. 

The pL-Aβ-Sup35MC and pU-Aβ-Sup35MC plasmids bearing *LEU2* and *URA3* selection markers, respectively, were constructed previously [37]. These plasmids carry the chimeric *Aβ-SUP35MC* gene under the control of the Copper-inducible *CUP1* promoter that is used to compensate for the deletion of the *SUP35* chromosomal copy [37]. The YGPM19p21 plasmid from the YSC4613 genomic library (Open Biosystems) bears the fragment of the yeast XVI chromosome containing the intact *SWI1* gene and has the *LEU2* selection marker. The pYCH-U2 centromeric plasmid contains full-length *SUP35* under the control of its endogenous promoter and *URA3* selection marker. 

The standard liquid and solid yeast cultural media (complete YEPD, or minimal MD) were used [45,46]. The cells were grown at 30 °C. For the transcriptome analysis, the cells were pre-grown in 5 mL of liquid MD medium supplemented with necessary amino acids, nitrogen bases, vitamins, and microelements containing 2% glucose as the carbon source until reaching OD_600nm_ = 0.5. Then, the cells were centrifuged (3000× *g*, 5 min) and inoculated into the 25 mL of pre-induction MD medium containing 2% raffinose instead of glucose. When the OD_600nm_ reached 0.5, the cells were centrifuged (3000× *g*, 5 min), inoculated into the 50 mL of MD containing 2% galactose as the sole carbon source, and were grown for 4 h. For the qPCR experiments, the cells were grown on the solid MD plates containing glucose or galactose as the sole carbon source in the indicated number of 24 h passages. 

For the nonsense suppression assay, yeast colonies were grown on the MD plates for 48 h, replica-plated on the MD plates without adenine or tryptophan, and grown for five days at 30 °C. To decrease the translation efficiency, aminoglycoside antibiotic paromomycin was added to the cultural media at the indicated concentrations. The growth of the strains on the plates containing galactose as the carbon source was analyzed after three replica-plating of the colonies with the duration of passage in 24 h [35].

### 2.2. DNA Preparation and qPCR

Genomic DNA for qPCR was extracted from the yeast cells using the MagJET Genomic DNA Kit (Thermo Fisher Scientific, Waltham, MA, USA). The sequences of primers and TaqMan qPCR probes are listed in Table S1. In each qPCR experiment, five independently obtained samples of DNA were tested. The ANK-32 real-time cycler (Institute of Analytical Instrumentation, Moscow, Russia) and BioRad CFX (BioRad, Hercules, CA, USA) were used. The results were processed using the 2^−ΔΔ*C*t^ method [47]. At the initial step, Δ*C*t that corresponds to the difference between the signal intensities of experimental and reference (*ACT1*) genes was calculated. Next, ΔΔ*C*t, indicating the difference between Δ*C*t-s in the experimental and control samples, was calculated. Finally, the mean of 2^−ΔΔ*C*t^, demonstrating the difference in the amounts of DNA in the experimental and control samples, was obtained. The significance of the differences observed was analyzed with the nonparametric Kruskal-Wallis test in R software (R Foundation, Vienna, Austria).

### 2.3. RNA Preparation and Whole Transcriptome RNA Sequencing (RNA-Seq)

Total RNA was extracted from the yeast cells using TRIzol LS Reagent (Invitrogen, Carlsbad, CA, USA), according to the manufacturer’s protocol. The concentration of RNA was calculated using the Quantus fluorimeter and QuantiFluor RNA System kit (Promega, Fitchburg, WI, USA). The quality control was performed with QIAxcel Advanced System (Qiagen, Hilden, Germany) capillary gel electrophoresis. The lower threshold for RIS quality control of the samples was no less than 5. The RNA libraries were prepared with the NEBNext Ultra Directional RNA Library Prep Kit for Illumina (New England BioLabs, Ipswich, MA, USA), NEBNext Poly(A) mRNA Magnetic Isolation Module (New England BioLabs), and oligonucleotide indexing set NEBNext Multiplex Oligos for Illumina, Index Primers Set 1 (New England BioLabs) from 1 µg of the total RNA.

The whole transcriptome RNA sequencing (RNA-Seq) was performed with the HiSeq 2500 sequencing platform (Illumina, San Diego, CA, USA) in the paired-end mode and with a read length of 2 × 100 bp using the TruSeq Rapid PE Cluster Kit—HS (Illumina) and TruSeq Rapid SBS Kit—HS (200 cycles) (Illumina).

### 2.4. RNA-Seq Read Processing, Pseudoalignment, and Data Analysis

Raw data comprising four biological by two technical replicates for each condition were processed as follows. First, the human contamination-related reads were eliminated with the *remove_human* script from the BBTools [48] package (version 38.16). The remaining reads underwent adapter trimming and quality filtering via the BBDuk trimming script from BBTools. The quality control of trimming was held with the FastQC software [49] (version 0.11.7). K-trimming was performed at the rightmost side of the reads with the k parameter equal to 27 and the quality trimming was performed at both sides with the minimal average quality per read equal to 30. The reads, having passed both operations, comprised 70% to 75% of the initial data, with the average read length of 200 bases. After that, the technical repeats for each biological sample were merged according to the read orientation. All technical information on library size and trimming is summarized in Table S2.

The resulting data were pseudoaligned to the reference protein-coding cDNA collection of the *S. cerevisiae* strain S288C via Kallisto [50] (version 0.44.0) in the pair-end mode with the number of bootstrap replicates equal to 100. Abundance tables produced at this stage were included in the analysis performed using the R Sleuth package [51] (version 0.30.0). All three biological conditions in question were compared to each other in a pairwise manner using both the likelihood ratio and the Wald post-hoc tests. The genes demonstrated a q-value (*p*-value undergone Benjamin-Hochberg multiple comparison adjustment [52]) of less than 0.001 in both tests, which were considered to be significantly regulated and included in a further analysis, with the genes possessing a β value (approximate natural log of fold change) of more than zero being treated as upregulated and those with a β value of less than zero being treated as downregulated. As for the *swi1*Δ to [*SWI*^+^] comparison, all significant results were divided into several specific groups according to their expression rate change in [*swi*^−^] to [*SWI*^+^] and [*swi*^−^] to *swi1*Δ tests, respectively. 

### 2.5. GO Term Overrepresentation Test

The subsets of up- and downregulated genes in both [*swi*^−^] to [*SWI*^+^] and [*swi*^−^] to *swi1*Δ comparisons were tested for GO overrepresented terms via the topGO [53] R package. The gene-to-term mapping was downloaded from the *Saccharomyces* Genome Database website [54] and processed manually to fit the package function requirements. The gene names for the respective subsets were tested against a gene universe comprising all *S. cerevesiae* gene names with the Fisher hypergeometric test and the ‘weight01’ graph reduction algorithm with the *p*-value cutoff baseline of 0.01. For the genes misregulated in the *swi1*Δ condition, some additional tests with chromosome I genes excluded both from the gene universe and the query were performed in order to assess whether chromosome duplication affects both differential expression and DEG set functional enrichment.

The resulting list of GO terms was exported as both data frames and topGO graph objects, and the latter were then merged by shared vertices and visualized using KEGGGraph [55] and igraph [56] R packages. In the case of graphs illustrating Biological Process and Molecular Function ontology enrichment, several vertices standing for internal non-significant nodes, as well as several reticular edges, were reduced for the sake of representativeness. 

### 2.6. KEGG Pathway Mapping

The respective sets of DEGs were also used for the KEGG Pathway test using the clusterProfiler [57] R package. Mapping was performed via the overrepresentation test, with *p*-values adjusted according to the Benjamin-Hochberg multiple test adjustment and q-value cutoff of 0.01. For each pathway in each assay, an enrichment ratio was calculated as the rate of genes mapped onto the respective pathway to all genes from *S. cerevisiae* related to this pathway. The resulting data were visualized with the ggplot2 [58] R package. Pathways of particular interest were also visualized and exported with the pathview R package [59] with a color scheme similar to that used for GO enrichment visualization. 

## 3. Results

### 3.1. Transcriptome-Wide Effects of the Swi1 Prion Formation and Deletion of its Structural Gene are Not Equal

**Figure 1:**
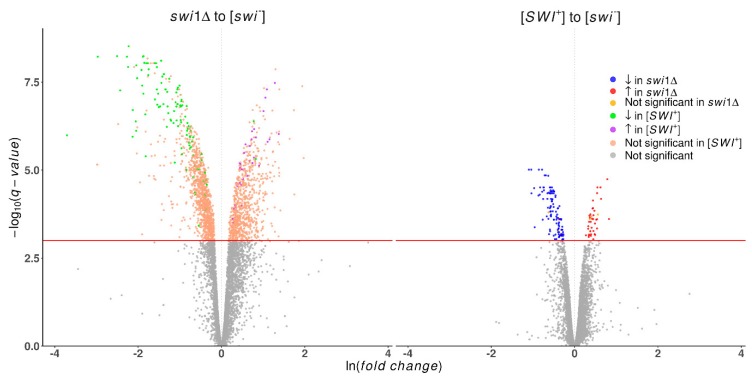
Volcano plots demonstrating differentially expressed genes in the *swi1*Δ and [*SWI*^+^] compared with [*swi*^−^] strains. Plotted on the *x*-axis is the ln fold difference between [*swi*^−^] and the corresponding strain. Plotted on the *y*-axis is the −log10(q-value) calculated with R package Sleuth [51]. Genes whose expression changes are nonsignificant are shown in gray below the red line, while significant differentially expressed genes (FDR < 0.001) are divided into categories, as follows. On the plot comparing *swi1*Δ to [*swi*^−^], green indicates genes significantly downregulated in [*SWI*^+^], purple represents genes significantly upregulated in [*SWI*^+^], and pink signifies genes which are nonsignificant in the comparison of [*SWI*^+^] to [*swi*^−^]. On the plot of [*SWI*^+^] to [*swi*^−^], genes downregulated in *swi1*Δ are shown blue, those upregulated in *swi1*Δ are red, and those which are nonsignificant in the comparison of *swi1*Δ to [*swi*^−^] are yellow.

A pairwise comparison of the control [*swi*^−^] and [*SWI*^+^] and *swi1*Δ states revealed a dramatic impact of the *SWI* gene deletion on the transcriptome, whilst the effect of prion appeared to be far less extensive (Table 1). We also performed a *swi1*Δ to [*SWI*^+^] comparison to elucidate some particular points of difference between these two conditions. As a result, 1139 differentially expressed genes were found, of which 90 genes were assumed to be artifacts of the used approach and excluded from further analysis as they did not appear in any of the experiment-to-control comparisons. The remaining genes from all three tests were processed into 10 groups, depending on the direction and magnitude of expression alteration (Table S3). These data basically imply changes caused by prion appear to simulate those triggered by deletion, but on a smaller scale (Figure 1). However, there were four upregulated genes (*ICS2*, *DLD3*, *PER33*, *ENA1*) and three downregulated genes (*IDP2*, *FMP16*, *AGX1*) in the prion state whose expression was not affected in the deletion state, as well as two genes (*HSP12*, *HXT5*) that were downregulated in the [*SWI*^+^] state and upregulated in *swi1*Δ. These genes encode proteins with different functions, part of which is related to the stress response (Table S4). In cases of concordant alteration expression, the change in deletion-affected strains usually had a notably higher magnitude, as estimated by the β parameter. 

**Figure 2:**
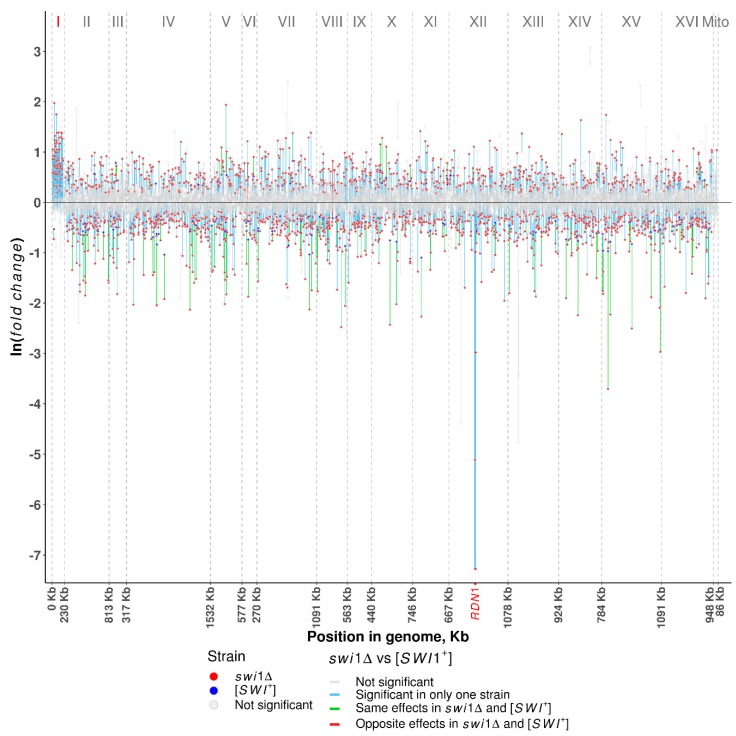
Distribution of differentially expressed genes along the chromosomes. Plotted on the *y*-axis is the ln fold difference between the *swi1*Δ or [*SWI*^+^] and [*swi*^−^] strains. Plotted on the *x*-axis are genomic coordinates. Vertical dashed lines denote the borders of chromosomes with the length of chromosomes shown on the *x-*axis and the name of chromosomes shown in the upper part of the plot. Red dots denote the significant (FDR < 0.001) changes in the *swi1*Δ and blue dots denote the significant changes in the [*SWI*^+^] strains. Grey dots denote nonsignificant changes in all strains. Lines connecting changes in the same genes, are (i) grey when changes in both *swi1*Δ and [*SWI*^+^] strains are nonsignificant, (ii) blue when change is significant in only one strain (*swi1*Δ or [*SWI*^+^]), (iii) green when a gene is up- or downregulated in both *swi1*Δ and [*SWI*^+^] strains, and (iv) red when a gene is upregulated in one strain and downregulated in another.

To estimate the reach of the observed effect across the yeast genome, we allocated differentially expressed genes to respective chromosomes (Figure 2, Table S5). In both cases, differential expression (both up- and downregulated) is distributed more or less uniformly across the genome, with the [*SWI*^+^] prion upregulating less than 1% and downregulating 0.7–3.1% of genes per chromosome and *SWI1* deletion affecting 6.9–14.4% and 15.3–18.5% of genes per chromosome, respectively (Table S5). The notable exception of this trend is a massive expression alteration of the genes in chromosome I occurring only on the *swi1*Δ background and resulting in the upregulation of 64 genes (~50%) and downregulation of only one gene (Table S5). These genes, except for the only repressed candidate, *BDH1*, are not differentially expressed in a prion-containing strain, leading one to conclude that the activation of genes in chromosome I may serve as a response to SWI/SNF impairment and be caused by the increase in the chromosome I copy number. Another specific feature of the *swi1*Δ state is the utter repression of several genes located on chromosome XII between bases 469,317 and 489,341 (Figure 2), either within or in close proximity to the *RDN1* locus containing multiple tandem copies of the rDNA repeats [60]. These genes comprise the *ASP3* paralogue family and five genes encoding putative proteins and share a β value ranging from −2.9 to −7.27, demonstrating the repression of this chromosomal region. However, the equal β value within the paralogous groups indicates that some of these results might be drawbacks of ambiguous alignment. Additionally, both [*SWI*^+^] and *swi1*Δ states do not actually affect expression of the mitochondrial genes, except for the *SCEI* gene upregulated on the deletion background.

### 3.2. SWI1 Deletion Causes the Chromosome I Disomy

**Figure 3:**
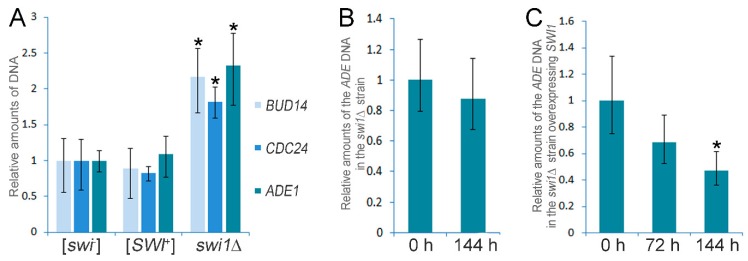
qPCR verification of the Chromosome I disomy in the *swi1*Δ strain and analysis of its stability and elimination. (**A**) Comparative qPCR analysis of the *ADE1*, *BUD14*, and *CDC24* genes copy number in the [*SWI*^+^], [*swi*^−^], and *swi1*Δ strains is shown. (**B**) Analysis of the stability of the Chromosome I disomy in the *swi1*Δ strain (*ADE1* was used as the marker of the chromosome I copy number). (**C**) The effects of the *SWI1* expression in the *swi1*Δ strain on the Chromosome I copy number (*ADE1* was used as the copy number marker). In all experiments, qPCR was performed using genomic DNA of the corresponding strain as the template and primers and probes listed in the Table S1. Yeast cells were grown on the complete media at 30 °C for the indicated time. The *ACT1* gene was used as a control. The results are shown as the 2^−ΔΔ*C*t^ ± the standard deviation. The means of the 2^−ΔΔ*C*t^ in the control samples or at the initial points of the experiments were set as 1. Five biological repeats were obtained for each experiment. The significance of the differences observed was analyzed with the nonparametric Kruskal-Wallis test. Asterisk indicates *p* ≤ 0.01.

The deletion of *SWI1* was found to cause an increase in the expression of ~50% genes located on the chromosome I (Figure 2, Table S5). This mean was remarkably high for the chromosome I, since for other chromosomes, it varied from 6.9 to 14.4% of the total number of genes (Table S5). Based on these data, we hypothesized that the *SWI1* deletion could cause the chromosome I disomy. To clarify whether the chromosome I is duplicated in the *swi1*Δ strain, we extracted genomic DNA from the [*SWI*^+^], [*swi*^−^], and *swi1*Δ strains and performed a quantitative polymerase chain reaction (qPCR) using three genetic markers (*ADE1* presented by the *ade1-14*_UGA_ nonsense allele which was previously found to be upregulated in the *swi1*Δ strain [43], *BUD14*, and *CDC24*) located on the chromosome I. The results of the qPCR demonstrated that the copy numbers of all three genes, *ADE1*, *BUD14*, and *CDC24*, are approximately two times higher in the *swi1*Δ strain in comparison with the [*SWI*^+^] and [*swi*^−^] strains (Figure 3A), thus supporting the data obtained with RNA-Seq and confirming the chromosome I duplication in the *swi1*Δ strain.

Next, we tested the stability of the chromosome I maintenance on the background of the *SWI1* deletion. We grew the *swi1*Δ cells for 144 h on the complete medium (approximately 60 generations) and tested the chromosome I copy number after this period of cultivation. We found that the copy number of the *ade1-14* remained the same after 144 h of cultivation (Figure 3B), showing that the chromosome I disomy is stably inherited in the cell divisions on the background of the *SWI1* deletion. 

An obvious question was whether the chromosome I disomy compensates for the possible inviability of the *SWI1* deletion previously reported for several yeast strains [61,62] or occurs independently on the deleterious effects of the Swi1 disturbance. We shuffled the pL-Aβ-Sup35MC plasmid to pU-Aβ-Sup35MC in the *swi1*Δ strain and re-introduced *SWI1* to this strain by transformation with the YGPM19p21 plasmid from the YSC4613 genomic library carrying the fragment of the XVI chromosome with the intact *SWI1* gene. After that, we analyzed the chromosome I copy number in the transformants after 72 h and 144 h of cultivation at 30 °C by the qPCR. The results demonstrated that after 72 h of cultivation (approximately 30 generations), the chromosome I copy number in the *SWI1*Δ strain remained statistically the same as at the initial point of experiment (Figure 3C). Nevertheless, having passed 60 generations (144 h), the *swi1*Δ cells lost additional chromosome I and its copy number was approximately two times less than at the beginning (Figure 3C).

Thus, the *SWI1* deletion causes the chromosome I disomy on the genetic background of the haploid yeast strain analyzed, and this disomy probably compensates for the lethality of the *SWI1* deletion since it is stably inherited but rapidly vanishes after the re-introduction of the *SWI1* gene. Notably, the [*SWI*^+^] prion formation does not cause the chromosome I disomy in contrast to the effects of the *SWI1* deletion.

### 3.3. SWI1 Deletion Modulates a Higher Number of Biological Processes and Molecular Functions than the [SWI^+^] Prion

**Figure 4:**
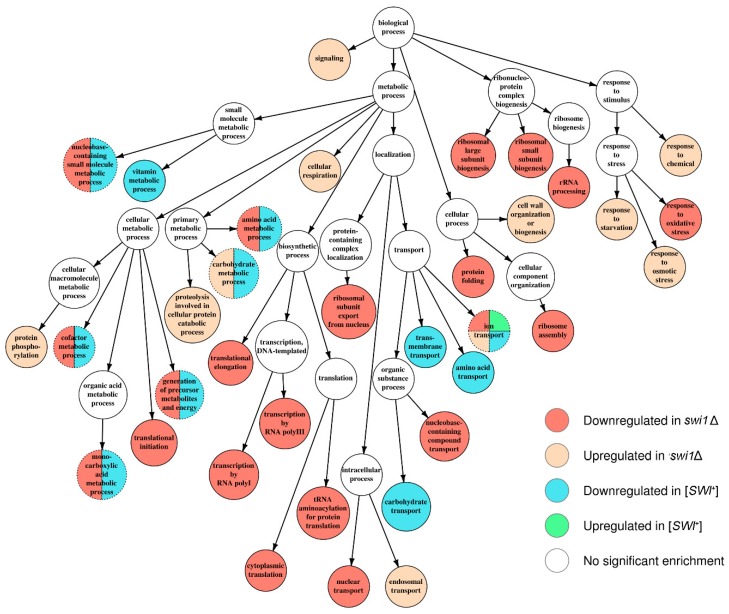
Graph of over-represented terms from Biological Process (BP) Gene Ontology. Several internal nodes and reticular edges were deleted to improve representativeness. For a full resolution image, see Figure S1.

**Figure 5:**
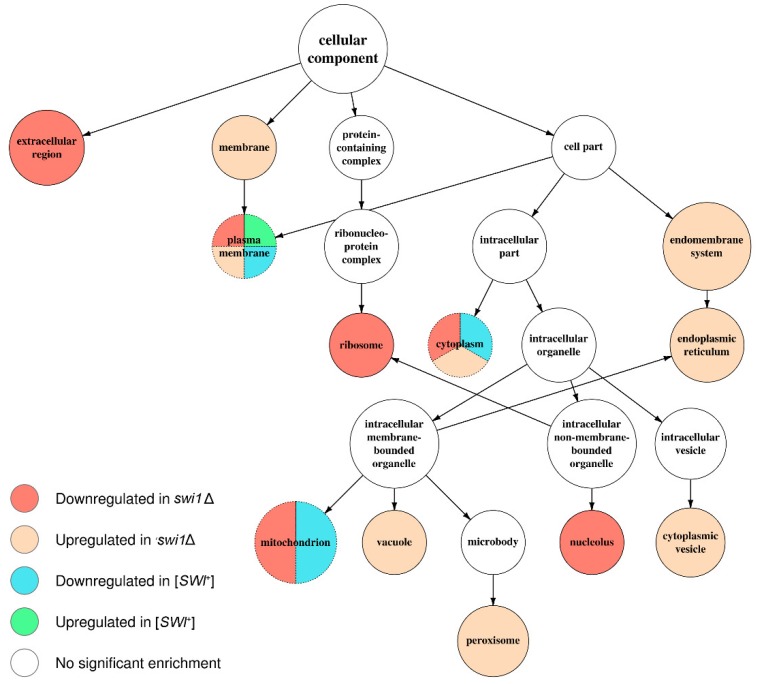
Graph of over-represented terms from Cellular Component (CC) Gene Ontology. Several internal nodes and reticular edges were deleted to improve representativeness. For a full resolution image, see Figure S2.

**Figure 6:**
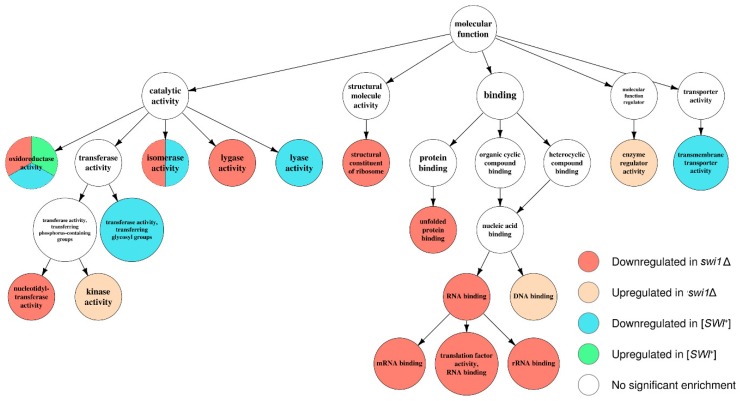
Graph of over-represented terms from Molecular Function (MF) Gene Ontology. For a full resolution image, see Figure S3.

In order to specify the effects of the observed transcriptional changes on cell physiology, we performed an annotation of several acquired sets of differentially expressed genes. We started with testing the sets standing for [*SWI*^+^] to [*swi*^−^] and *swi1*Δ to [*swi*^−^] comparisons for over-represented Gene Ontology terms using the topGO package for R language (Table S6). The resulting terms were then mapped onto shared term hierarchy graphs depicting three primal ontologies, namely the Biological Process (BP), the Cellular Component (CC), and the Molecular Function (MF) (Figure 4, Figure 5 and Figure 6, Figures S1–S3, respectively). In addition, such analysis was then repeated for *swi1*Δ to [*swi*^−^] DEGs, but with the genes located on the chromosome I excluded to avoid possible bias introduced by an adaptive duplication in the deletion-affected samples; this, however, resulted in slight changes in GO mapping of the repressed gene subset (addition of one term per both BP and MF ontology mapping and exclusion of only one term from BP mapping and two terms from CC mapping, respectively, see Table S6).

Apparently, the deletion-regulated gene subset appeared to be more enriched in various terms than the subset from the prion-containing strain. Several terms, most of which comprise BP ontology (Figure 4), are shared between the deletion-related and the prion-related DEGs, and might be related to phenotypic similarities between respective samples. However, most of the enriched terms were not shared between conditions, except for the most common terms, such as ‘ion transport’ from BP (Figure 4) and ‘plasma membrane’ from CC (Figure 5). One of the most notable traits of the deletion-affected samples is the downregulation of genes related to different stages of mRNA translation. The terms related to the translation are especially over-represented in the BP graph (Figure 4) and include rRNA processing and ribosome biogenesis and export to cytoplasm. To add some evidence, this correlates with the downregulation of ‘ribosome’ and ‘nucleolus’ terms in the CC graph (Figure 5) and ‘structural constituent of ribosome’ and cluster of terms inferior to ‘RNA binding’ in the MF graph (Figure 6). 

Moreover, the translation impairment affects not only ribosome functionality, but also translational initiation and elongation (Figure 4); it is also noteworthy that ‘nucleotidyl-transferase activity’ from MF ontology is also downregulated (Figure 6) which may, in this aspect, be related to aminoacyl-tRNA synthesis. Finally, the transcriptional activity of RNA polymerases I and III activities also seem to be disrupted by the deletion only (Figure 4). At the same time, the terms promoted by deletion are mostly related to various stress responses and vacuolar transport (Figure 4), which is supported by the upregulation of terms related to vacuoles and other membranous organelles (Figure 5). Unlike this, the [*SWI*^+^] prion formation does not affect either transcription or translation and results in downregulation of the genes attributed to the GO terms related to transmembrane transport of various small metabolites, as well as their biogenesis and metabolism (Figure 4 and Figure 6), while the genes whose expression increases in the presence of the prion show very poor enrichment in any of the three ontologies (Figure 4, Figure 5 and Figure 6).

### 3.4. The Effects of SWI1 Deletion and [SWI^+^] Prion on Metabolic Pathways Correlate with Their Phenotypic Manifestations

**Figure 7:**
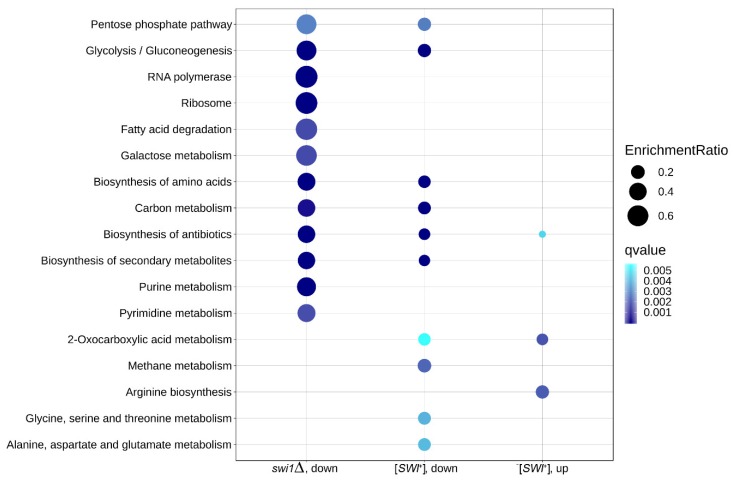
Over-represented KEGG Pathway term found in this work. Dot size represents the enrichment ratio, and color gradient depicts the experimental q-value in the respective assay. Pathways are sorted in descendant order based on the total enrichment ratio in all three categories.

To validate the obtained results and to specify their effect on particular metabolic and functional pathways, we then performed a KEGG Pathway overrepresentation test on down- and upregulated genes in each condition (Figure 7). Surprisingly, no overrepresented pathways were found in the deletion-promoted genes. In the other three subsets, 17 pathways were found to be overrepresented overall, with seven pathways present in at least two subsets. Among these acquired pathways, several were found to be ‘global’ metabolic pathways and thus were dismissed as non-significant, and the rest were visualized via the Pathview R package for a closer look (Figure S4). The genes were mapped for both experimental conditions, even if in one of them, the pathway was not found to be over-represented. Deterioration of the carbohydrate metabolic pathways, such as the glycolysis and pentose phosphate pathway, seems to be concordant between prion- and deletion-affected samples, though the *SWI1* deletion replenishes glucose dissimilation with the upregulation of genes recruiting alternative carbon sources to CoA acetylation (Figure S4A,B). At the same time, the galactose metabolism appears to be downregulated exclusively in the deletion state (Figure S4C). Another overrepresented trait of deletion is massive downregulation of the nucleobase synthesis (Figure S4D,E), considering both major biosynthesis pathways and reactions leading to nucleobase-containing cofactors and other nitrogen-containing compounds. The purine synthesis impairment is also partially reproduced in the [*SWI*^+^] samples, though the repression there mostly affects downstream reactions, such as inter-nucleobase transmutation rather than major pathways. In addition, [*SWI*^+^] tends to both repress and promote specific amino acid metabolic pathways. Despite the fact that the ‘biosynthesis of amino acids’ pathway was overrepresented in the set of genes repressed by the *SWI1* deletion, none of the specific pathways were found by the overrepresentation test for the deletion-affected samples, so we mapped these genes onto metabolic pathways acquired for prion-regulated genes, as well as tryptophan biosynthesis, which could be related to the observed phenotype (Figure S4F–H). Surprisingly, prion and deletion showed a similar effect on arginine biosynthesis, with four genes concordantly upregulated and one gene downregulated (Figure S4G). All these results concur with the overrepresented terms obtained for the BP GO ontology for different amino group-containing moieties, including both nucleobase-containing metabolites and amino acids.

Speaking of previously observed translational inactivation on the *swi1*Δ background, only one related pathway, namely ‘ribosome’, was found to be over-represented (Figure S4I). Indeed, most of the ribosomal proteins present in yeast appear to be deficient in the *swi1*Δ strain. However, this term solely describes the ribosome constituent, while GO testing revealed a complex negative impact of deletion on the translation machinery. Following this surmise, we collected all translation-related KEGG pathways and mapped all DEGs onto them (Figure S4J–M). Apparently, all the terms are notably impacted by the deletion, while the prion-regulated genes do not appear in the graphs, and that deletion causes the visible activation of several genes involved alongside mostly repressive effects. This mapping allows tracking down a cumulative effect of the *SWI1* deletion on almost all stages of translation and protein biogenesis, including ribosome biogenesis and export from the nucleus (Figure S4J), mRNA processing and nonsense-mediated decay (Figure S4K), export to the cytoplasm (Figure S4L), and aminoacyl-tRNA synthesis (Figure S4M). 

**Figure 8:**
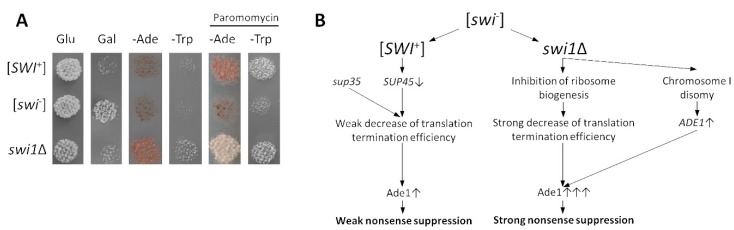
Nonsense suppression and mechanisms of its development in the [*SWI*^+^] and *swi1*Δ strains. (**A**) Deletion of *SWI1* causes omnipotent nonsense suppression, even in the presence of wild type *SUP35*, while the addition of aminoglycoside antibiotic paromomycin leads to nonsense suppression in both the [*SWI*^+^] and *swi1*Δ strains. Images of –Ade and –Trp plates were obtained after five days of incubation at 30 °C. Images of MD plates with glucose (Glu) and galactose (Gal) as the sole carbon sources were taken after 24 h of incubation at 30 °C (before this, Gal plate was three times replica plated with duration of passages in 24 h). (**B**) Scheme illustrating molecular mechanisms of the nonsense suppression in the [*SWI*^+^] and *swi1*Δ strains (for details, see Discussion).

Since the RNA-Seq data demonstrated that the deletion of *SWI1* caused general repression of the translation-related processes, while [*SWI*^+^] did not lead to these effects, we checked whether this difference can be detected phenotypically. The [*SWI*^+^] prion was initially identified in our strains as a weak omnipotent suppressor of the nonsense alleles *ade1-14*_UAG_ and *trp1-289*_UGA_ [37]. This causes translational read-through that leads to omnipotent nonsense suppression, resulting in the growth of the [*SWI*^+^] strain on the media without adenine or tryptophan, respectively. This effect is phenotypically detected only in the presence of the mutant variants of *SUP35* encoding release factor eRF3 with decreased functional activity [35]. Here, we tested the phenotypes of the [*SWI*^+^] and *swi1*Δ strains in which a weak suppressor *SUP35* variant Aβ-Sup35MC was substituted for a wild-type *SUP35* under the control of its endogenous promoter located on the pYCH-U2 plasmid. As expected, the [*SWI*^+^] and *swi1*Δ strains exhibited the same growth on the MD plates with glucose as the sole carbon source, but demonstrated strong growth inhibition on the MD plates with galactose as the sole carbon source (Figure 8A). Surprisingly, the *swi1*Δ strain exhibited strong nonsense suppression on the media without adenine and tryptophan, reflecting global disturbance of translation occurring in the presence of *SWI1* deletion, while [*SWI*^+^] did not exhibit this effect (Figure 8A). Nevertheless, addition to the cultural media of the aminoglycoside antibiotic paromomycin that has multiple binding sites in the eukaryotic ribosome and inhibits almost all stages of protein synthesis [63], thus mimicking the *SWI1* deletion effects, caused the appearance of the same suppressor phenotype in the [*SWI*^+^] strain (Figure 8A), confirming the crucial role of the ribosome biogenesis defect in developing the phenotypic manifestation of the *SWI1* deletion.

## 4. Discussion

The comparative analysis of the transcriptomic effects of the prion formation and deletion of the structural gene of the Swi1 protein performed in this study demonstrated that both the [*SWI*^+^] prion and *SWI1* deletion modulate the expression of hundreds of genes on the media containing galactose as the sole carbon source (Table 1). These changes affect various molecular functions, biological processes, and metabolic pathways (Figure 4, Figure 5, Figure 6 and Figure 7), reflecting pleiotropic phenotypic manifestations of the [*SWI*^+^] prion and the *SWI1* deletion described in various studies (Table 2). Despite some general similarities between the subsets of genes affected by these two states of *SWI1*, the effects of *swi1*Δ are much stronger than [*SWI*^+^] and involve at least two highly specific effects that do not arise in the presence of the prion: (i) the chromosome I disomy and (ii) the repression of the ribosome biogenesis and translation, including the downregulation of almost all genes for ribosomal proteins, as well as genes located on the chromosome XII within rDNA locus *RDN1*. 

The repression of the locus *RDN1* encoding rRNA demonstrated (in this work) for the *SWI1* deletion was previously found to be also caused by the deletion of *SNF2* encoding another component of the SWI/SNF chromatin remodeler [64]. Moreover, we found that not only the expression of *RND1* is affected by the *SWI1* deletion, but the genes encoding ribosomal proteins, the translation factors, and biosynthesis of most of the aminoacyl-tRNAs are also downregulated on such a background (Figure S4). The downregulation of the translation-related genes was also demonstrated for the *SNF2* and *SWI3* mutants [30,65]. Thus, inactivation of at least three subunits of SWI/SNF affects ribosome biogenesis, suggesting the role of this complex as the essential regulator of translation. Moreover, at least three of the four SWI/SNF modules [30] (catalytic (presented by Snf2), regulatory (Swi3), and Swi1 module) are involved in the expression of translation-related genes. Interestingly, the deletions of peripheral subunits of SWI/SNF (Snf5 and Snf12) did not affect translational machinery [30]. Thus, *SWI1* is likely to be important for the core function of SWI/SNF, at least in the regulation of the translation-related genes.

Notably, the prion formation by *SWI1* does not cause a significant decrease of the translation-related genes, which can be easily monitored by the growth of the [*SWI*^+^] strains containing respective nonsense mutations (*ade1-14*_UGA_ and *trp1-289*_UAG_) on the media that lacked adenine or tryptophan (nonsense suppression) (Figure 8A). We found that the *SWI1* deletion causes strong omnipotent nonsense suppression, even in the presence of the wild-type Sup35 (eRF3) (Figure 8A), while [*SWI*^+^] causes nonsense suppression either in the presence of mutant Sup35 with decreased eRf3 functional activity [35,42] or on the media with aminoglycoside antibiotics [40] that block different stages of translation [63], imitating the effects of the *SWI1* deletion. Taking together, in contrast to [*SWI*^+^], the deletion of the *SWI1* gene causes global inhibition of translation that is detected phenotypically.

The SWI/SNF remodelers are known to be essential for genome stability, not only as master regulators of transcription, but also via maintaining sister chromosome cohesion and the DNA damage response, at least in mammals [66]. We found that *SWI1* deletion causes a stably inherited chromosome I disomy on the genetic background of the 11-1-1-D931 yeast strain, but an additional chromosome I copy gradually disappears when the wild-type *SWI1* is re-introduced, suggesting a compensatory effect of such a disomy (Figure 3). A similar effect was previously found in several [*PSI*^+^] (a prion of Sup35) strains [67] that also exhibited a strong nonsense suppressor phenotype [68]. Thus, the chromosome I disomy could play an important role in compensation of the defects in translation termination fidelity. Nevertheless, the *SWI1* deletion also causes downregulation of the purine biosynthesis (Figure S4). Thus, the chromosome I disomy may potentially alleviate this process by increasing *ade1-14* levels, which was previously found to compensate for the growth defects of the strains with the same genotype on a media without adenine [43]. Notably, the aneuploidy was demonstrated to be associated with the formation of different non-chromosomal determinants affecting the translation termination efficiency, like [*ISP*^+^] (initially described as a prion form of the transcriptional regulator Sfp1 [11] and later connected with the chromosome II copy number [69]), as well as the [*ASP*^+^] (chromosome VIII disomy [70]). Probably, the prion formation by several proteins regulating key biological processes could induce genome instability in a few fractions of cells that are inherited by progeny under selective pressure and determine adaptive phenotypic traits like resistance to poisonous compounds. 

Another feature of the chromosome I disomy, proteotoxic stress caused by the impairment of protein quality-control pathways, resulted in intense protein aggregation in the cytoplasm, which was described in [71]. In our study, *swi1*Δ yeast was marked by abundant expression misregulation of genes related to protein folding control (*HSP10*, *HSP12*, *HSP26*, *HSP30*, *HSP42*, and *HSP104*), ubiquitination (*UBP5*, *UBP11*, and *UBP13*), and proteasome biogenesis and proteolytic activity (*RPN4-9*, *HUL5*, *SPG5*, *POC4*, *SEM1*, *HSC82*, *SRP1*, and several more), thus indirectly backing up the notion about the deteriorating effect of the chromosome I disomy on proper folding supervision. Furthermore, most of these effects were not registered in [*SWI*^+^] cells’ transcriptome. 

The molecular processes underlying even similar phenotypic manifestations of the [*SWI*^+^] and *SWI1* deletion are different. In the case of the suppression of *ade1-14*, [*SWI*^+^] causes a decrease in the eRF1 (*SUP45*) amounts that leads to the phenotypically detected nonsense suppression only in the presence of the mutant eRF3 variants, with decreased functional activity [35,42] (Figure 8B). In contrast, the *SWI1* deletion causes both the general inhibition of translation-related genes and overexpression of *ade1-14* by the chromosome I disomy that leads to nonsense suppression, even in the presence of wild-type *SUP35* (Figure 8 B). The inhibition of the translation-related genes seems to be a primary source of nonsense suppression since *trp1-289* is suppressed in the *swi1*Δ strains expressing wild-type *SUP35*, and this effect can be reached in the [*SWI*^+^] strain by the addition of aminoglycosides (Figure 8B). The deletion of the *SWI1* blocks induction of the *GAL* regulon essential for the galactose metabolism [72,73] (Figure S4), thus leading to the inhibition of growth on the media containing galactose as the sole carbon source, while [*SWI*^+^] causes the same effect, but does not affect the expression of *GAL* genes (Figure S4). Galactose is involved in the energy production by either a consequent transmutation into lactose and then α-d-glusose or an exchange between α-d-galactose phosphate and UDP-glucose [74]; in both cases, the glucose-derived product is then recruited to glycolysis. This might shed light on the nature of this phenocopy as most of the glycolitic enzymes are concordantly repressed in both [*SWI*^+^] and *swi1*Δ samples (Figure S4). 

Another feature of *swi*Δ cells is the altered response to various stress conditions (Figure 4). The recent findings suggest that SWI/SNF plays an important role in the stress response. Indeed, the Snf2 catalytic subunit of SWI/SNF was shown to be recruited to response of the genes under osmotic stress by means of the Cyc8-Tup1 regulatory network [75], and this recruitment is positively affected by kinases of the PKA family [76]. In addition, the sets of genes misregulated by deletions of *SWI2* and *TAF14* that encode the transcriptional regulator involved in the stress alleviation overlap significantly [77]. However, it does not explain why most of the response types are positively modulated in the *swi1*Δ cells, while the oxidative stress response is repressed. On the latter, one might suggest a link to positive regulation of the ‘cellular respiration’ term (Figure 4) and thus interdependence between the repression of the oxidative stress response genes and the activation of the oxidative phosphorylation genes. To date, the connection between SWI/SNF activity and ROS formation has been found in human cells, where inactivation of the ARID1B SWI1-like protein leads to the deterioration of the oncogene-induced senescence of malignant cells, a process associated with ROS formation [78]. 

It is noteworthy that at least seven genes were found to be altered in their expression in [*SWI*^+^] yeast, though they remained intact on the *swi1*Δ background. Of these, four genes (*ICS2*, *DLD3*, *PER33*, and *ENA1*) were activated and three (*IDP2*, *FMP16*, and *AGX1*) were repressed by the prion. In addition, *HSP12* and *HXT5* were downregulated in the [*SWI*^+^] cells and upregulated in *swi1*Δ. Respective proteins seem to share nothing in common in their functions; however, further inspection showed that all of these genes fall under regulation by the Oaf1 transcription factor either directly or via downstream transcription regulators. The Oaf1 protein is involved in the control of fatty acid β-oxidation, peroxisome formation, and telomeric silencing, and is encoded by a gene located in chromosome I [79,80]. This gene is also upregulated in *swi1*Δ cells, presumably by means of dose compensation. Probably, compensatory chromosome I disomy leading to *OAF1* upregulation in the *SWI1*Δ but not [*SWI*^+^] cells might partially alleviate the effects of Swi1 inactivation.

Taken together, both the [*SWI*^+^] prion and the *SWI1* deletion cause global changes in the expression of the yeast genome. Despite overall similarity between the subsets of the genes whose expression is modulated by [*SWI*^+^] and *swi1*Δ and their phenotypic manifestations, the impact of *SWI1* deletion on the modulation of gene expression is significantly much stronger and involves specific effects. These effects of *swi1*Δ include: (i) the general downregulation of the translation-related genes; (ii) the chromosome I disomy; and (iii) different patterns of up- and downregulation of various biological processes, molecular functions, and metabolic pathways, including the downregulation of *GAL* regulon and purine metabolism. In contrast, [*SWI*^+^] does not cause such a repression of the main metabolic pathways and processes and specifically modulates the expression of several genes. Thus, [*SWI*^+^] prion formation exhibits only partial loss-of-function effects, with several gain-of-function features. These data suggest that yeast prions are modulators of functions of normal cellular proteins rather than pathological ‘dead ends’ of their misfolding and aggregation. 

## Figures and Tables

**Table 1:** Transcriptomic effects of the [*SWI*^+^] prion and *SWI1* deletion on the media with galactose as the sole carbon source.

Changes in Gene Expression	Comparison		
	[*SWI*^+^] to [*swi*^−^]	*swi1*Δ to [*swi*^−^]	*swi*Δ to [*SWI*^+^]
Upregulated	40	822	409
Downregulated	119	1156	730
Total	159	1978	1139

**Table 2:** Similarities and differences between the effects of the [*SWI*^+^] prion and the *swi1* deletion.

Effect or Phenotype	Manifestation in the Strain Containing		Condition	Reference
	[*SWI*^+^] Prion	*SWI1* Deletion		
Decreased vegetative growth	+	+	Media with galactose, glycerol or raffinose as carbon source and Antimycin-A	[7]
Nonsense suppression in the presence of mutant *SUP35* (eRF3) variants	+	+	Media without adenine; in the presence of mutant eRF3 variants with decreased functional activity	[35,37,40,42]
Decreased vegetative growth	+	+	Media with galactose or glycerol as carbon source	[35]
Loss of flocculation	+	+	Stationary-phase cultures	[36]
Loss of invasiveness	+	+	Complete media, 6 days of incubation	[36]
Abolished pseudohyphal growth	+	+	SLAD media containing 4% glucose	[36]
Repression of the *FLO1* and *FLO11* expression	+	+	Complete media with glucose as carbon source	[36]
Increased expression of the *ADE1* gene	-	+	Complete media	[43]
Chromosome I disomy	-	+	Complete media	This study
Inhibition of the ribosome biogenesis and translation	-	+	Complete media with galactose as carbon source	This study
Omnipotent nonsense suppression in the presence of wild-type *SUP35* (eRF3)	-	+	Media without adenine or tryptophan; in the presence of the wild-type eRF3	This study
Decreased expression of the *SUP45* (eRF1) gene	+	-	Complete media with glucose as carbon source	[41,42]
Aggregation of the Mss11, Sap30, Gts1 and Msn1 transcriptional regulators	+	-	Overproduction of the YFP-fused proteins under the *GAL1* promoter	[36]

## Supplementary Materials

The following are available online at https://www.mdpi.com/2073-4425/10/3/212/s1, Table S1: Oligonucleotides used in this study, Table S2: RNA-Seq library sizes before and after trimming and technical repeat merging, Table S3: Genes whose expression levels are affected by the [*SWI*^+^] prion or *SWI1* deletion, Table S4: Genes whose expression is specifically modulated by the [*SWI*^+^] prion, Table S5: Distribution of differentially expressed genes across chromosomes and mtDNA, Table S6: Biological processes, molecular functions, and cellular components that are affected by the [*SWI*^+^] prion or *SWI1* deletion, Figure S1: Biological Process Graph in *svg* format, Figure S2: Cellular Component Graph in *svg* format, Figure S3: Molecular Function Graph in *svg* format, Figure S4: Visualized KEGG pathways with DEG mapping from [*SWI*^+^] and *swi1*Δ.
