# Supplementary material for: RNA Sequencing Reveals Specific Transcriptomic Signatures Distinguishing Effects of the [SWI+] Prion and SWI1 Deletion in Yeast Saccharomyces cerevisiae
Source: Genes (Basel). 2019 Mar 12;10(3):212. doi: 10.3390/genes10030212 (PMC6471900; doi:10.3390/genes10030212)
Supplement: Supplementary file 1 [file genes-10-00212-s001.zip › Figure_S4.pdf]

Figure S4. KEGG Pathway mapping of DEGs from [SWI+] and swi1Δ

- Downregulated in swi1 Δ
- Upregulated in swi1 Δ
- Downregulated in [SWI+]
- Upregulated in [SWI+]
- No significant expression alteration
- Absent in species of interest

A: Glysolysis and gluconeogenesis
B: Pentose phosphate pathway
C: Galactose metabolism
D: Purine metabolism
E: Pyrimidine metabolism
F: Alanine, aspartate and glutamate metabolism
G: Arginie biosynthesis
H: Tryptophan metabolism
I: Ribosome
J: Ribosome biogenesis in eukaryotes
K: mRNA surveillance pathway
L: RNA transport
M: Aminoacyl-tRNA biosynthesis

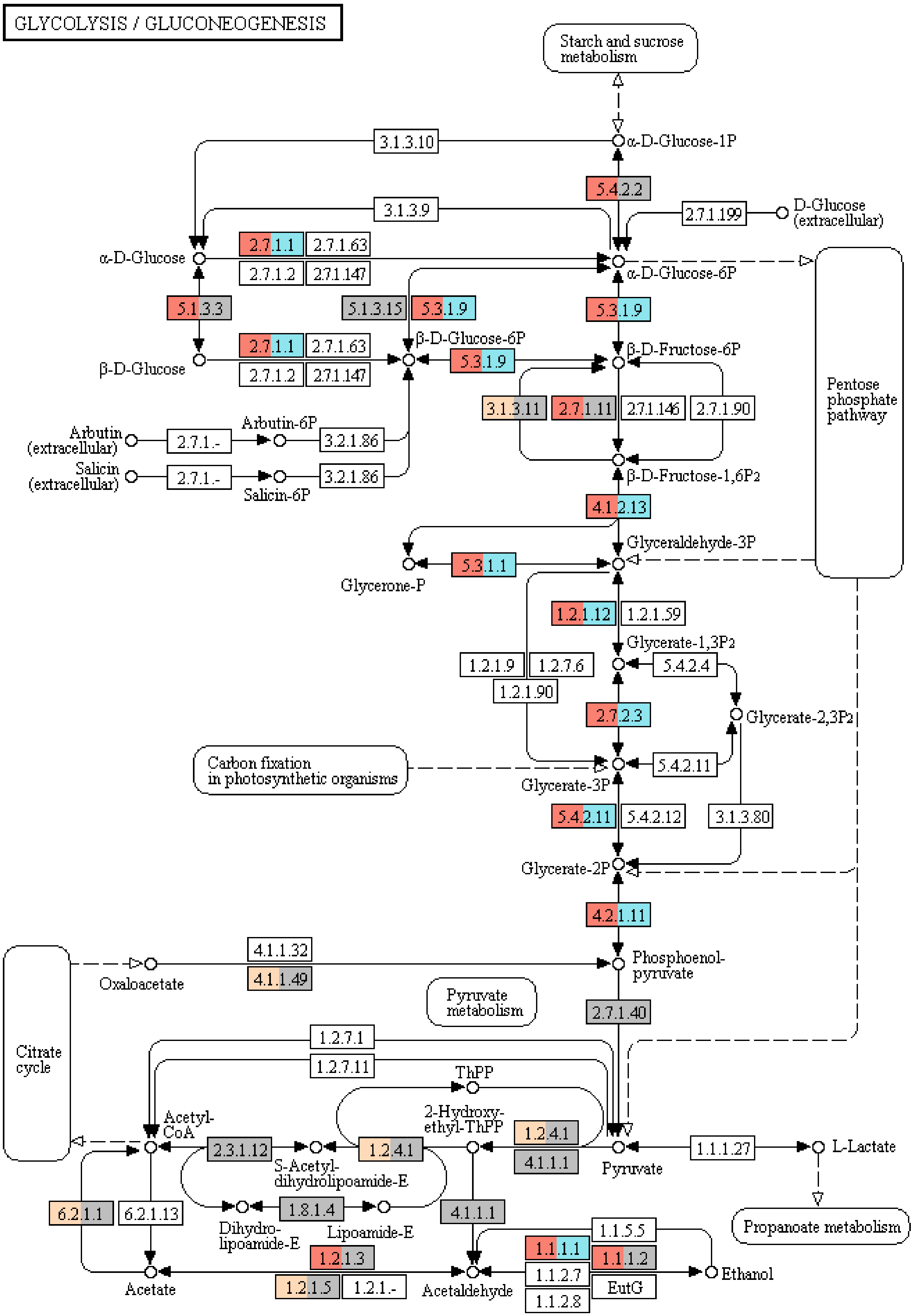

Data on KEGG graph
Rendered by Pathview

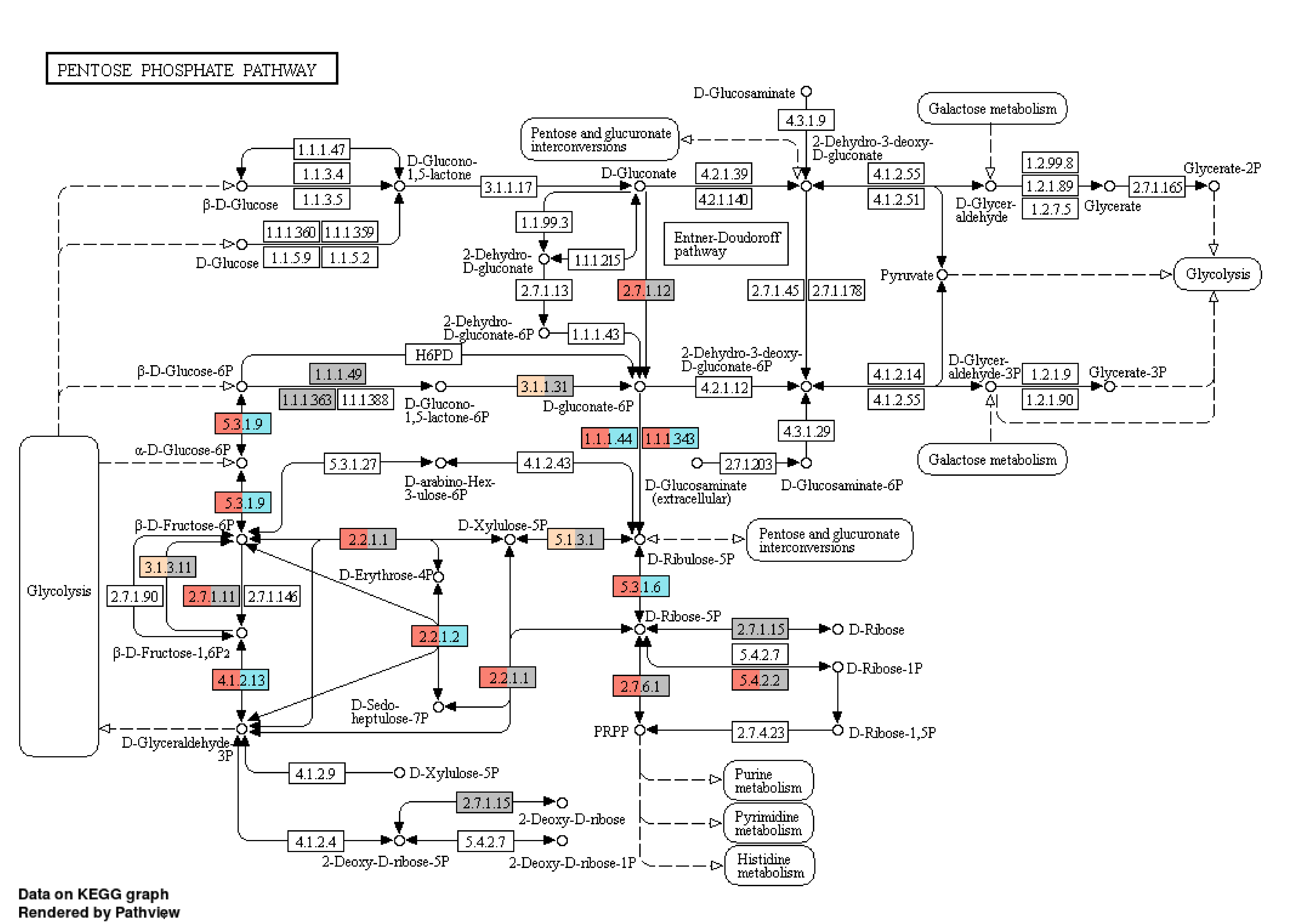
PENTOSE PHOSPHATE PATHWAY
D-Glucosaminate
4.3.1.9
Galactose metabolism
Pentose and glucuronate interconversions
2-Dehydro-3-deoxy-D-gluconate
1.1.1.47
D-Glucono-1,5-lactone
D-Gluconate
4.2.1.39
4.1.2.55
1.2.99.8
Glycerate-2P
1.1.3.4
3.1.1.17
4.2.1.140
4.1.2.51
1.2.1.89
2.7.1.165
β-D-Glucose
1.1.3.5
D-Glycer-aldehyde
1.2.7.5
Glycerate
1.1.99.3
1.1.1.360
1.1.1.359
Entner-Doudoroff pathway
D-Glucose
1.1.5.9
1.1.5.2
2-Dehydro-D-gluconate
1.1.1.215
Pyruvate
Glycolysis
2.7.1.13
2.7.1.12
2.7.1.45
2.7.1.178
2-Dehydro-D-gluconate-6P
1.1.1.43
H6PD
2-Dehydro-3-deoxy-D-gluconate-6P
D-Glycer-aldehyde-3P
β-D-Glucose-6P
1.1.1.49
3.1.1.31
4.1.2.14
1.2.1.9
Glycerate-3P
1.1.1.363
1.1.1.388
4.2.1.12
D-Glucono-1,5-lactone-6P
D-gluconate-6P
4.1.2.55
1.2.1.90
5.3.1.9
1.1.1.44
1.1.1.343
4.3.1.29
α-D-Glucose-6P
5.3.1.27
4.1.2.43
2.7.1.203
Galactose metabolism
D-arabino-Hex-3-ulose-6P
D-Glucosaminate (extracellular)
D-Glucosamine-6P
5.3.1.9
β-D-Fructose-6P
D-Xylulose-5P
2.2.1.1
5.1.3.1
Pentose and glucuronate interconversions
D-Ribulose-5P
3.1.3.11
D-Erythrose-4P
Glycolysis
2.7.1.90
2.7.1.11
2.7.1.146
5.3.1.6
D-Ribose-5P
2.7.1.15
D-Ribose
2.2.1.2
β-D-Fructose-1,6P2
5.4.2.7
D-Ribose-1P
2.2.1.1
4.1.2.13
5.4.2.2
2.7.6.1
D-Sedo-heptulose-7P
PRPP
2.7.4.23
D-Ribose-1,5P
D-Glyceraldehyde-3P
4.1.2.9
D-Xylulose-5P
Purine metabolism
2.7.1.15
2-Deoxy-D-ribose
Pyrimidine metabolism
4.1.2.4
5.4.2.7
2-Deoxy-D-ribose-5P
2-Deoxy-D-ribose-1P
Histidine metabolism
Data on KEGG graph
Rendered by Pathview

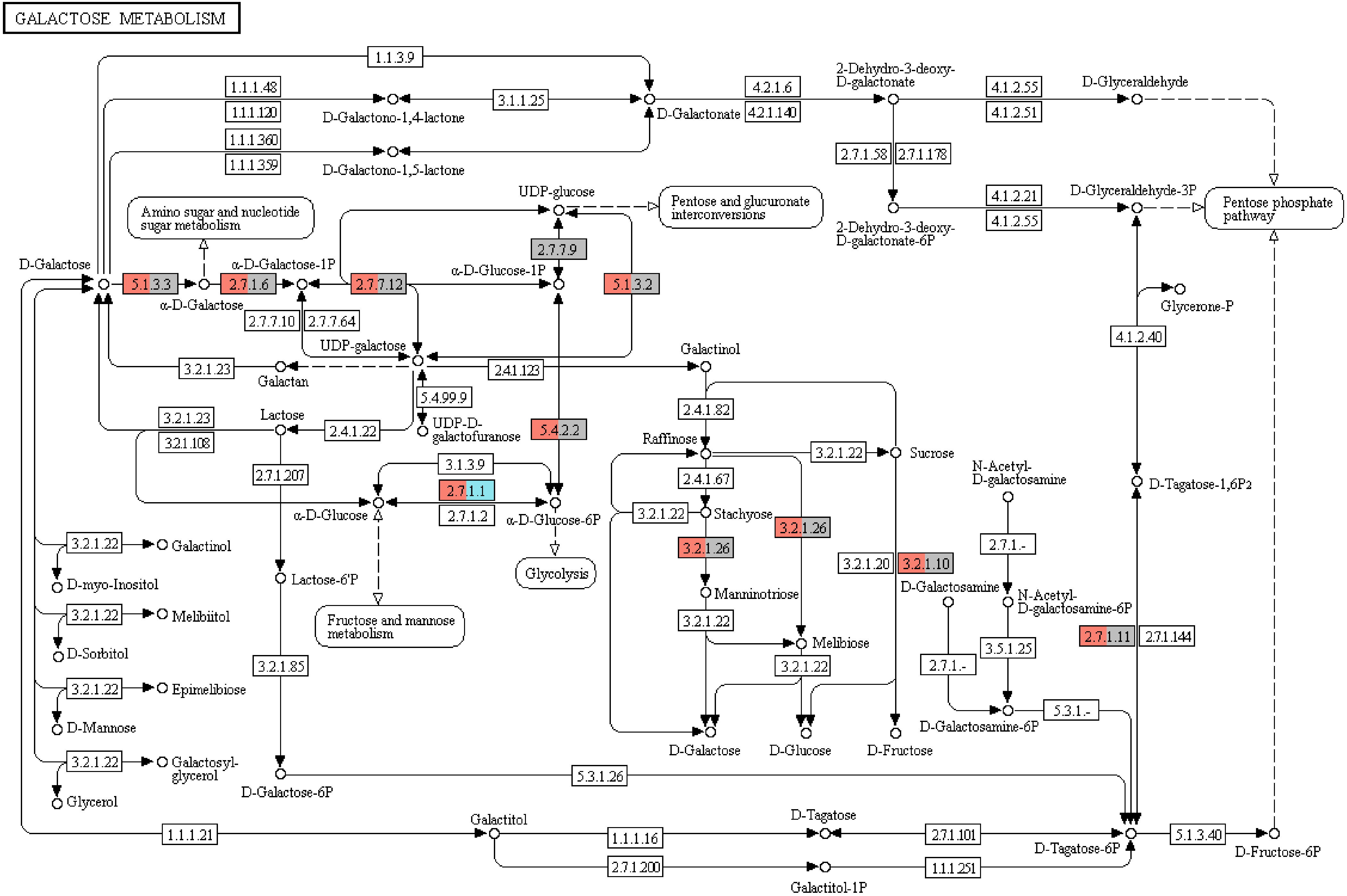

Data on KEGG graph
Rendered by Pathview

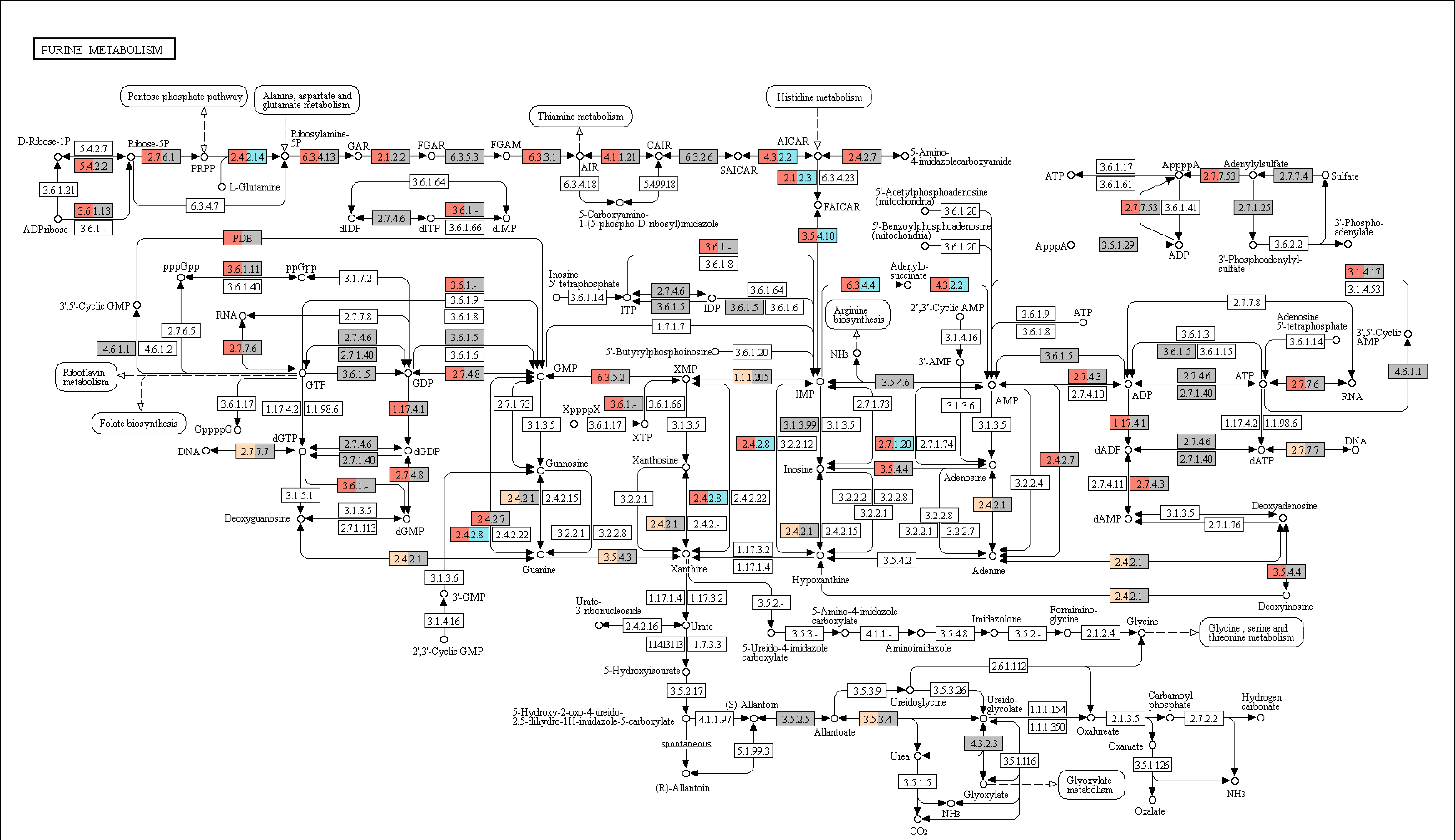

Data on KEGG graph
Rendered by Pathview

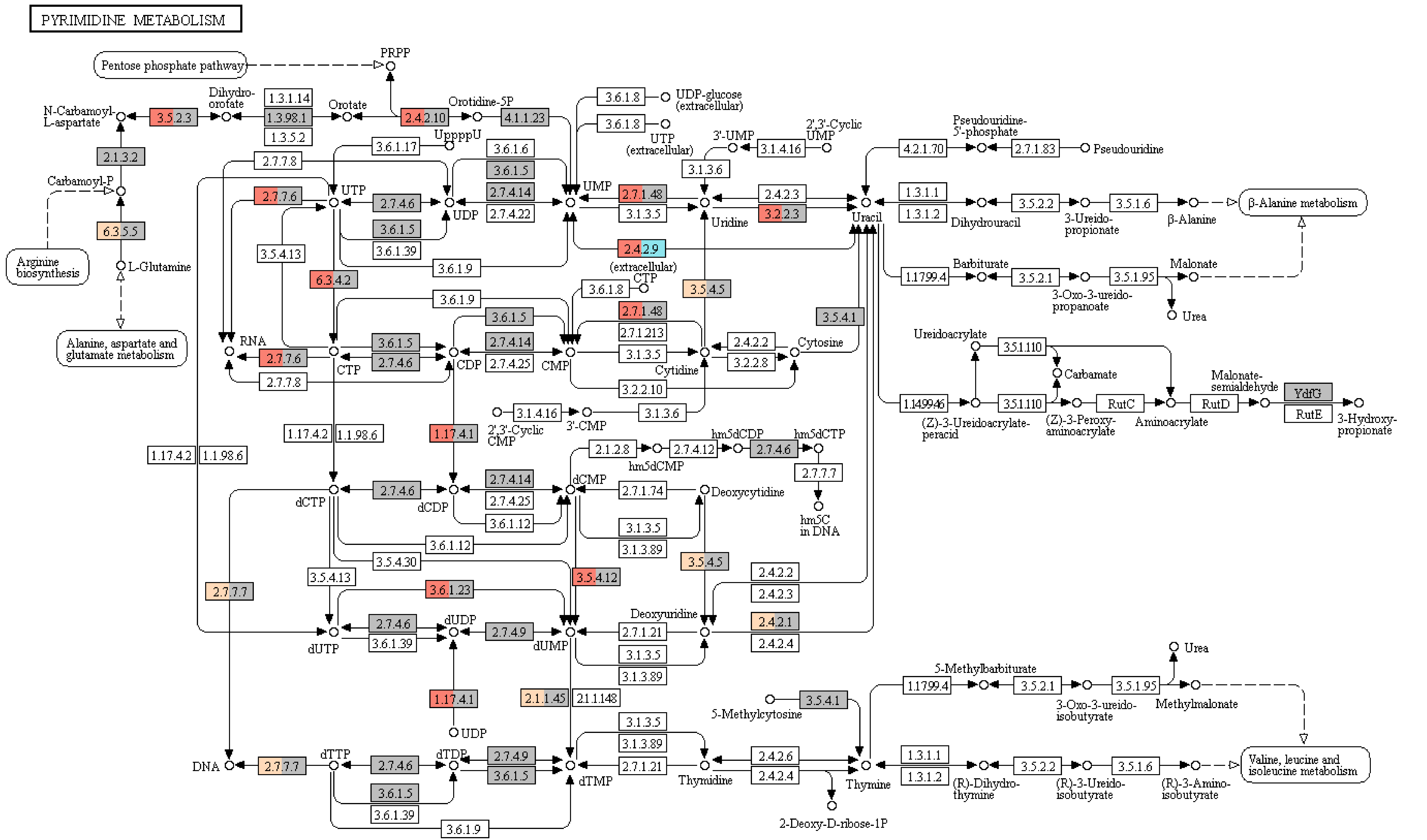

Data on KEGG graph
Rendered by Pathview

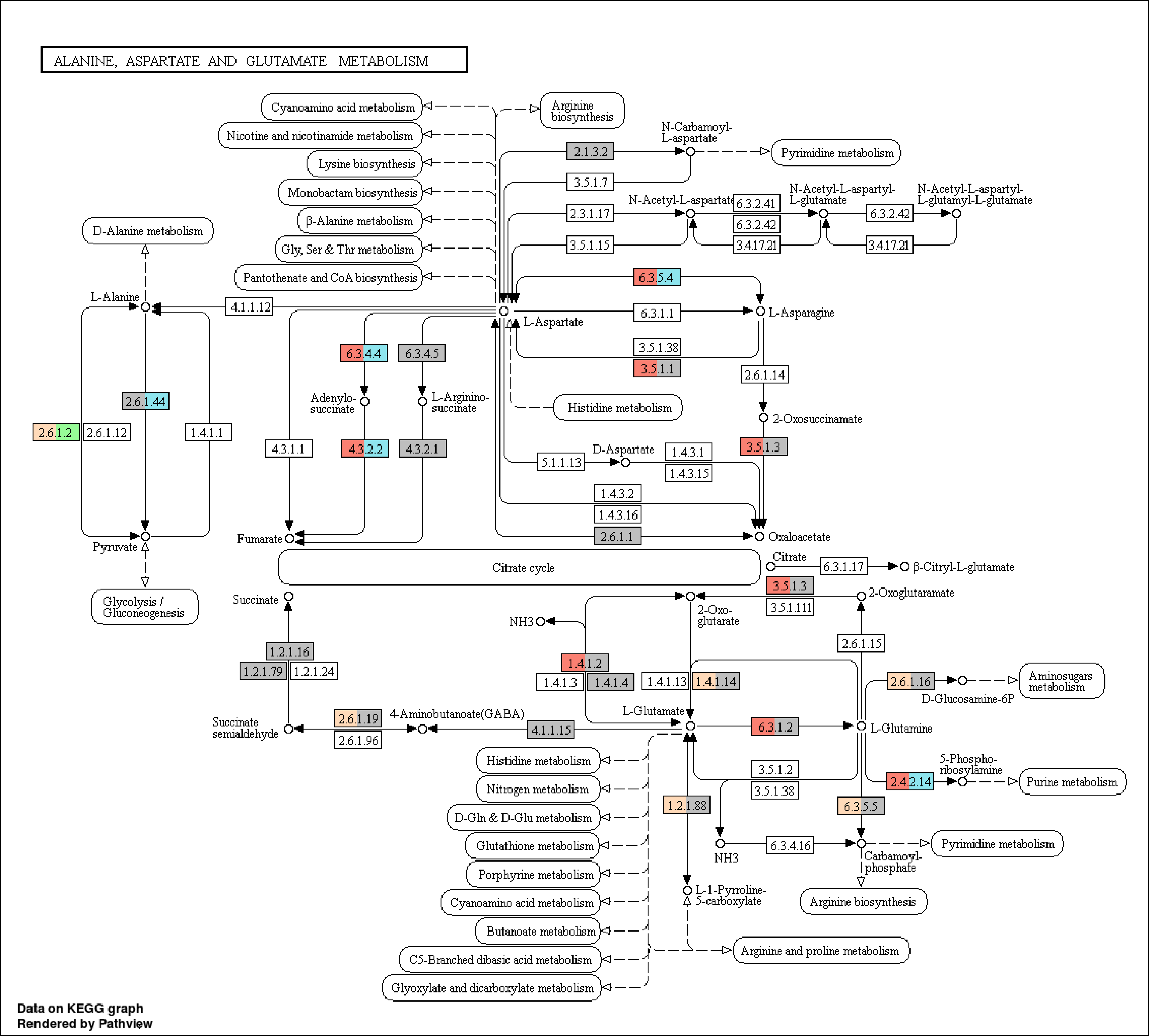
ALANINE, ASPARTATE AND GLUTAMATE METABOLISM
Cyanoamino acid metabolism
Nicotine and nicotinamide metabolism
Lysine biosynthesis
Monobactam biosynthesis
β-Alanine metabolism
Gly, Ser & Thr metabolism
Pantothenate and CoA biosynthesis
Arginine biosynthesis
D-Alanine metabolism
N-Carbamoyl-L-aspartate
Pyrimidine metabolism
2.1.3.2
3.5.1.7
2.3.1.17
3.5.1.15
N-Acetyl-L-aspartate
6.3.2.41
6.3.2.42
3.4.17.21
N-Acetyl-L-aspartyl-L-glutamate
N-Acetyl-L-aspartyl-L-glutamyl-L-glutamate
6.3.5.4
L-Alanine
4.1.1.12
L-Aspartate
6.3.1.1
L-Asparagine
3.5.1.38
3.5.1.1
6.3.4.4
6.3.4.5
2.6.1.44
2.6.1.2
2.6.1.12
1.4.1.1
Adenylo-succinate
L-Arginino-succinate
Histidine metabolism
2.6.1.14
2-Oxosuccinamate
3.5.1.3
4.3.1.1
4.3.2.2
4.3.2.1
5.1.1.13
D-Aspartate
1.4.3.1
1.4.3.15
1.4.3.2
1.4.3.16
2.6.1.1
Pyruvate
Fumarate
Oxaloacetate
Citrate cycle
Citrate
6.3.1.17
β-Citryl-L-glutamate
Glycolysis / Gluconeogenesis
Succinate
2-Oxoglutaramate
3.5.1.111
NH3
2-Oxo-glutarate
2.6.1.15
1.2.1.16
1.2.1.79
1.2.1.24
1.4.1.2
1.4.1.3
1.4.1.4
1.4.1.13
1.4.1.14
2.6.1.16
Aminosugars metabolism
D-Glucosamine-6P
Succinate semialdehyde
2.6.1.19
2.6.1.96
4-Aminobutanoate(GABA)
4.1.15
L-Glutamate
6.3.1.2
L-Glutamine
5-Phospho-ribosylamine
2.4.2.14
Purine metabolism
Histidine metabolism
Nitrogen metabolism
D-Gln & D-Glu metabolism
Glutathione metabolism
Porphyrine metabolism
Cyanoamino acid metabolism
Butanoate metabolism
C5-Branched dibasic acid metabolism
Glyoxylate and dicarboxylate metabolism
3.5.1.2
3.5.1.38
1.2.1.88
6.3.5.5
NH3
6.3.4.16
Carbamoyl-phosphate
Pyrimidine metabolism
Arginine biosynthesis
L-1-Pyrroline-5-carboxylate
Arginine and proline metabolism
Data on KEGG graph
Rendered by Pathview

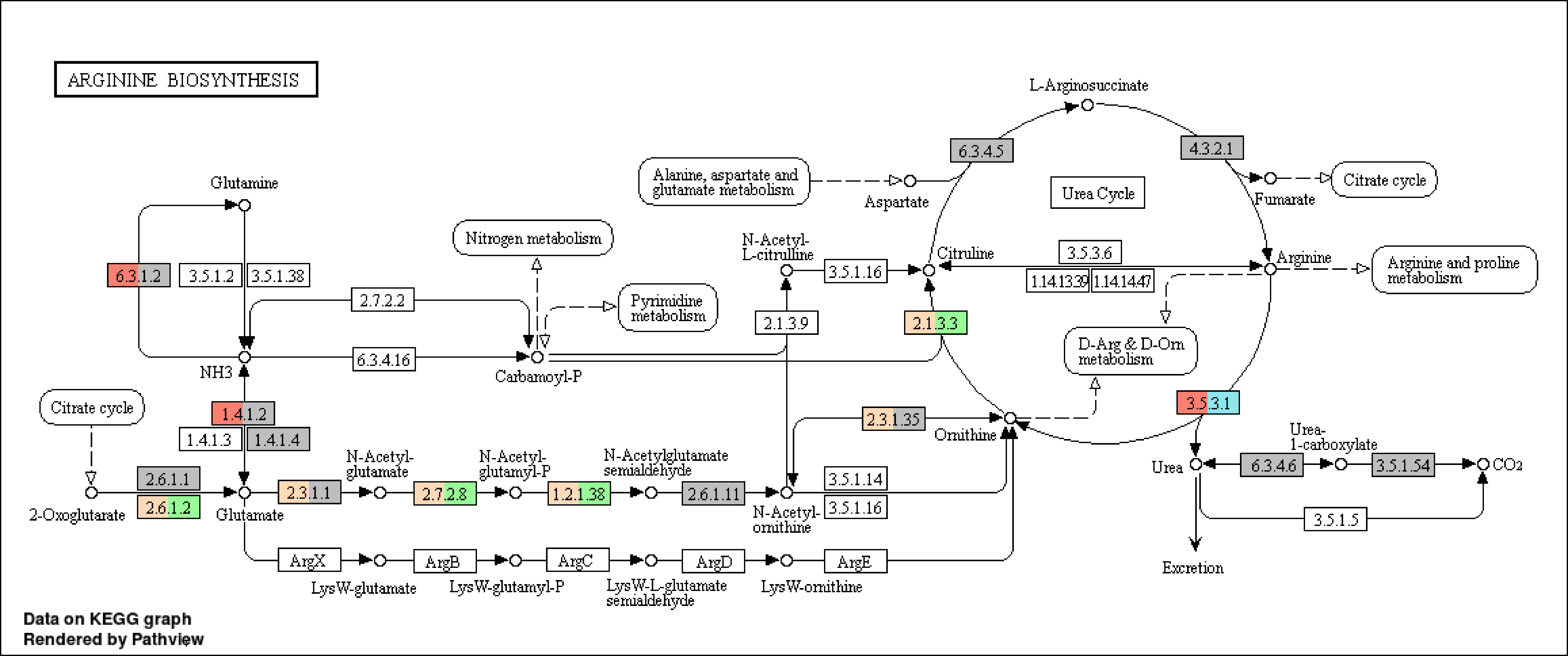

ARGININE BIOSYNTHESIS
L-Arginosuccinate
6.3.4.5
4.3.2.1
Alanine, aspartate and glutamate metabolism
Aspartate
Citrate cycle
Fumarate
Urea Cycle
Glutamine
Nitrogen metabolism
N-Acetyl-L-citrulline
Citruline
3.5.3.6
Arginine
Arginine and proline metabolism
6.3.1.2
3.5.1.2
3.5.1.38
3.5.1.16
1.14.13.39
1.14.14.47
2.7.2.2
Pyrimidine metabolism
2.1.3.9
2.1.3.3
D-Arg & D-Orn metabolism
NH3
6.3.4.16
Carbamoyl-P
Citrate cycle
1.4.1.2
3.5.3.1
1.4.1.3
1.4.1.4
2.3.1.35
Ornithine
Urea-1-carboxylate
Urea
6.3.4.6
3.5.1.54
CO2
N-Acetyl-glutamate
N-Acetyl-glutamyl-P
N-Acetylglutamate semialdehyde
2.6.1.1
3.5.1.14
2-Oxoglutarate
2.6.1.2
Glutamate
2.3.1.1
2.7.2.8
1.2.1.38
2.6.1.11
N-Acetyl-ornithine
3.5.1.16
3.5.1.5
Excretion
ArgX
ArgB
ArgC
ArgD
ArgE
LysW-glutamate
LysW-glutamyl-P
LysW-L-glutamate semialdehyde
LysW-ornithine
Data on KEGG graph
Rendered by Pathview

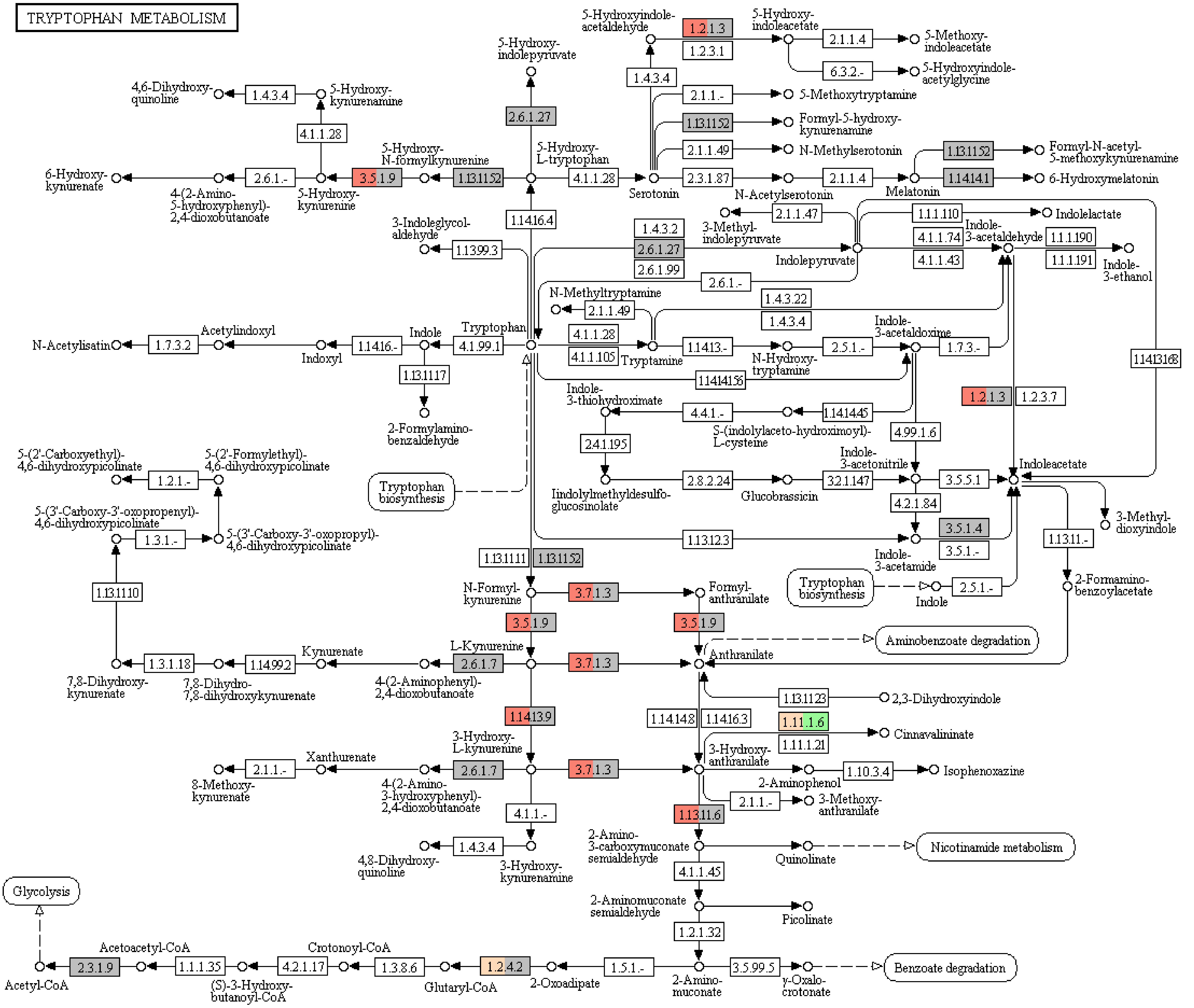

Data on KEGG graph
Rendered by Pathview

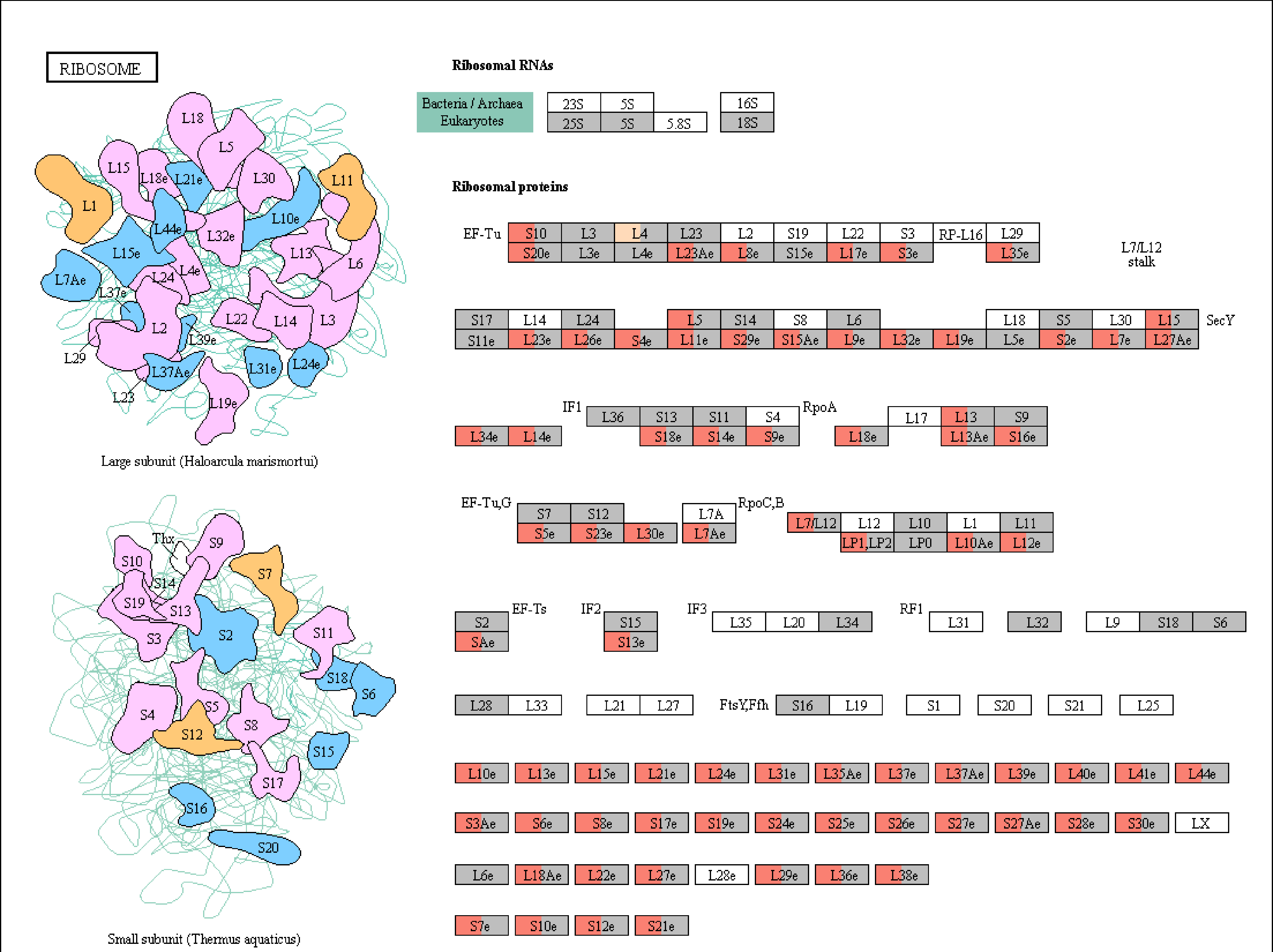

Data on KEGG graph
Rendered by Pathview

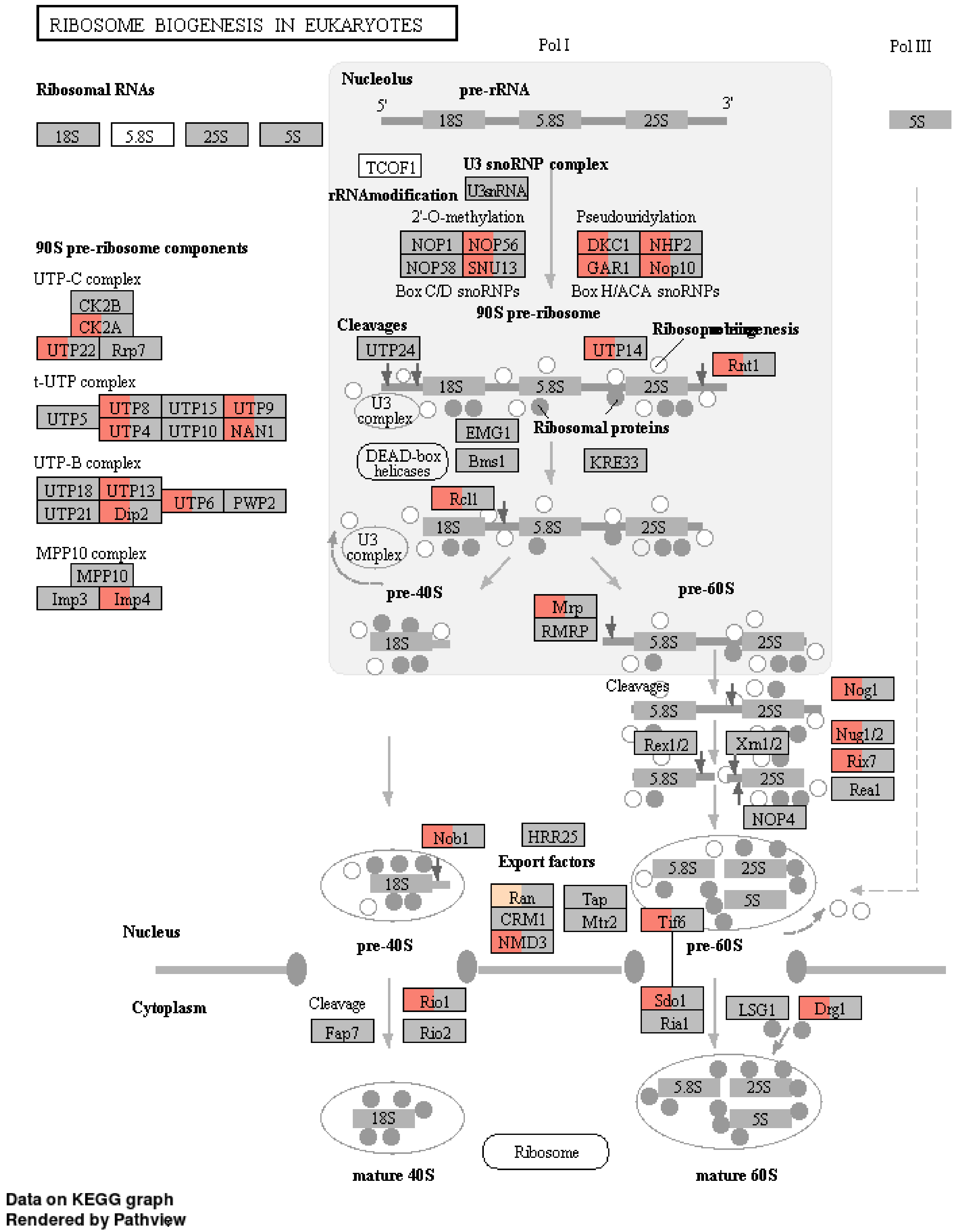
RIBOSOME BIOGENESIS IN EUKARYOTES
Pol I
Pol III
Ribosomal RNAs
18S
5.8S
25S
5S
Nucleolus
pre-rRNA
5'
3'
TCOF1
U3 snoRNP complex
U3snRNA
rRNAmodification
2'-O-methylation
Pseudouridylation
NOP1
NOP56
NOP58
SNU13
DKC1
NHP2
GAR1
Nop10
Box C/D snoRNPs
Box H/ACA snoRNPs
90S pre-ribosome
90S pre-ribosome components
UTP-C complex
CK2B
CK2A
UTP22
Rrp7
t-UTP complex
UTP5
UTP8
UTP15
UTP9
UTP4
UTP10
NAN1
UTP-B complex
UTP18
UTP13
UTP6
PWP2
UTP21
Dip2
MPP10 complex
MPP10
Imp3
Imp4
Cleavages
UTP24
UTP14
Rnt1
U3 complex
EMG1
Ribosomal proteins
DEAD-box helicases
Bms1
KRE33
Rcl1
pre-40S
pre-60S
Mrp
RMRP
Nog1
Nug1/2
Rix7
Rea1
Rex1/2
Xrn1/2
NOP4
Nob1
HRR25
Export factors
Ran
CRM1
NMD3
Tap
Mtr2
Tif6
Nucleus
Cytoplasm
Cleavage
Rio1
Fap7
Rio2
Sdo1
Ria1
LSG1
Drg1
mature 40S
Ribosome
mature 60S
Data on KEGG graph
Rendered by Pathview

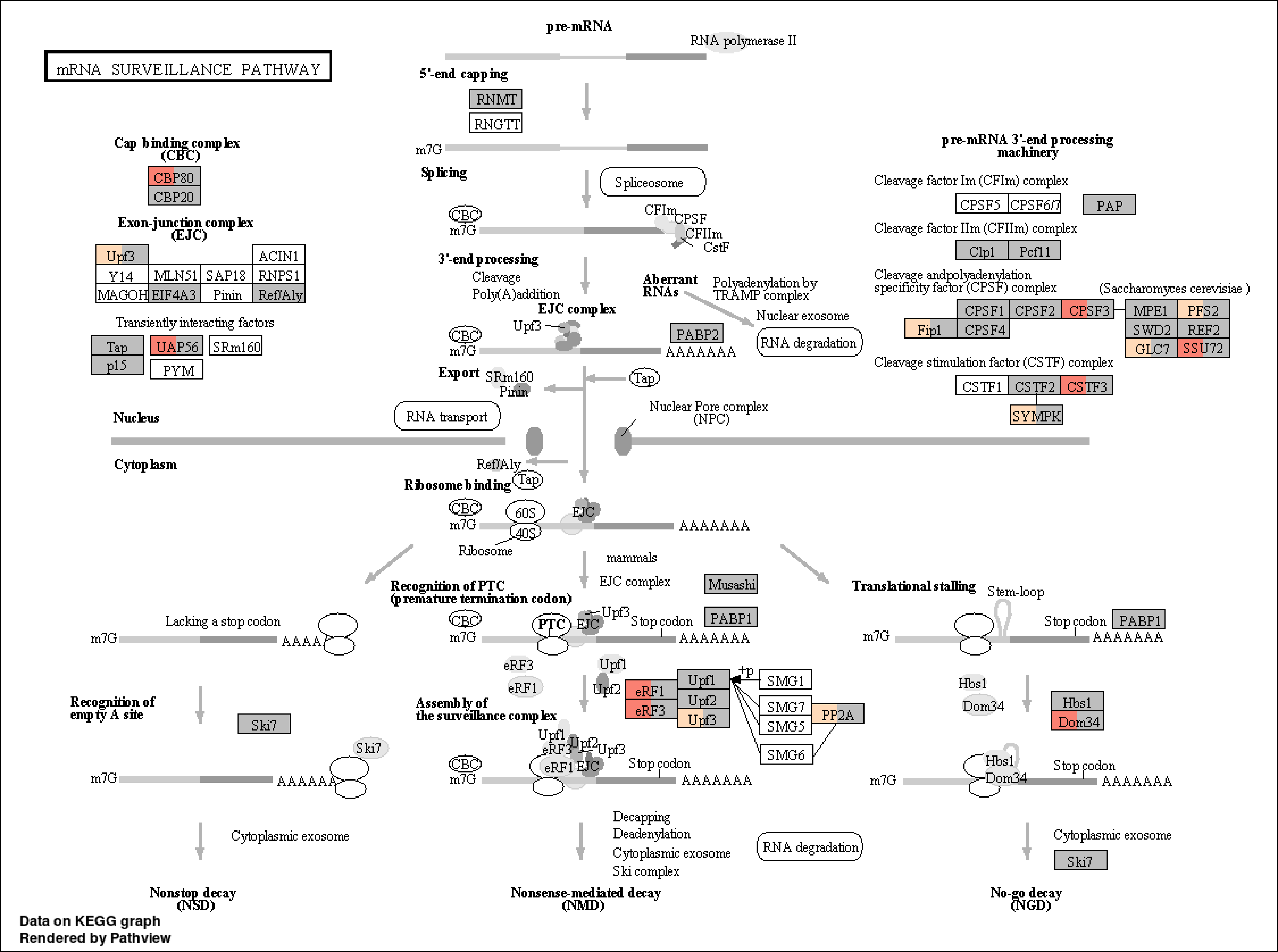
mRNA SURVEILLANCE PATHWAY
pre-mRNA
RNA polymerase II
5'-end capping
RNMT
RNGTT
m7G
Splicing
Spliceosome
CBC
CFIm
CPSF
CFIIm
CstF
3'-end processing
Cleavage
Poly(A)addition
EJC complex
Upf3
PABP2
AAAAAAA
Aberrant RNAs
Polyadenylation by TRAMP complex
Nuclear exosome
RNA degradation
Export
SRm160
Pinin
Tap
RNA transport
Nuclear Pore complex (NPC)
Nucleus
Cytoplasm
Ref/Aly
Ribosome binding
60S
40S
EJC
Ribosome
Cap binding complex (CBC)
CBP80
CBP20
Exon-junction complex (EJC)
Upf3
ACIN1
Y14
MLN51
SAP18
RNPS1
MAGOH
EIF4A3
Pinin
Ref/Aly
Transiently interacting factors
Tap
UAP56
SRm160
p15
PYM
pre-mRNA 3'-end processing machinery
Cleavage factor Im (CFIm) complex
CPSF5
CPSF6/7
PAP
Cleavage factor IIm (CFIIm) complex
Clp1
Pcf11
Cleavage andpolyadenylation specificity factor (CPSF) complex
(Saccharomyces cerevisiae)
CPSF1
CPSF2
CPSF3
MPE1
PFS2
Fip1
CPSF4
SWD2
REF2
GLC7
SSU72
Cleavage stimulation factor (CSTF) complex
CSTF1
CSTF2
CSTF3
SYMPK
mammals
EJC complex
Musashi
Recognition of PTC (premature termination codon)
PTC
Upf3
Stop codon
PABP1
Lacking a stop codon
AAAA
Translational stalling
Stem-loop
Stop codon
PABP1
eRF3
eRF1
Upf1
Upf2
eRF1
eRF3
Upf1
Upf2
Upf3
+p
SMG1
SMG7
SMG5
PP2A
SMG6
Assembly of the surveillance complex
Recognition of empty A site
Ski7
Hbs1
Dom34
Hbs1
Dom34
Ski7
AAAAAA
Upf1
eRF3
Upf2
Upf3
eRF1
EJC
Stop codon
Hbs1
Dom34
Stop codon
Cytoplasmic exosome
Decapping
Deadenylation
Cytoplasmic exosome
Ski complex
RNA degradation
Cytoplasmic exosome
Ski7
Nonstop decay (NSD)
Nonsense-mediated decay (NMD)
No-go decay (NGD)
Data on KEGG graph
Rendered by Pathview

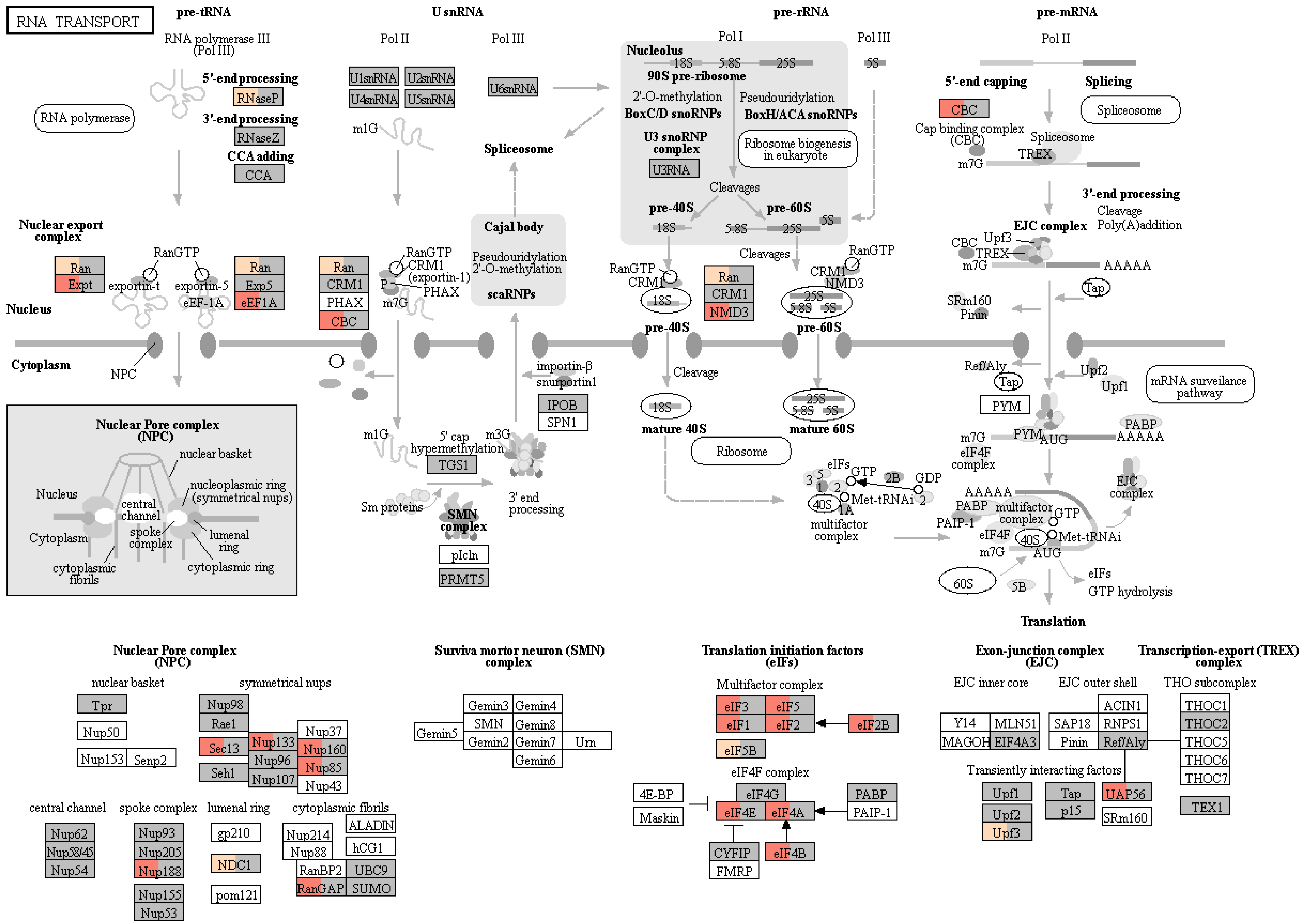

Data on KEGG graph
Rendered by Pathview

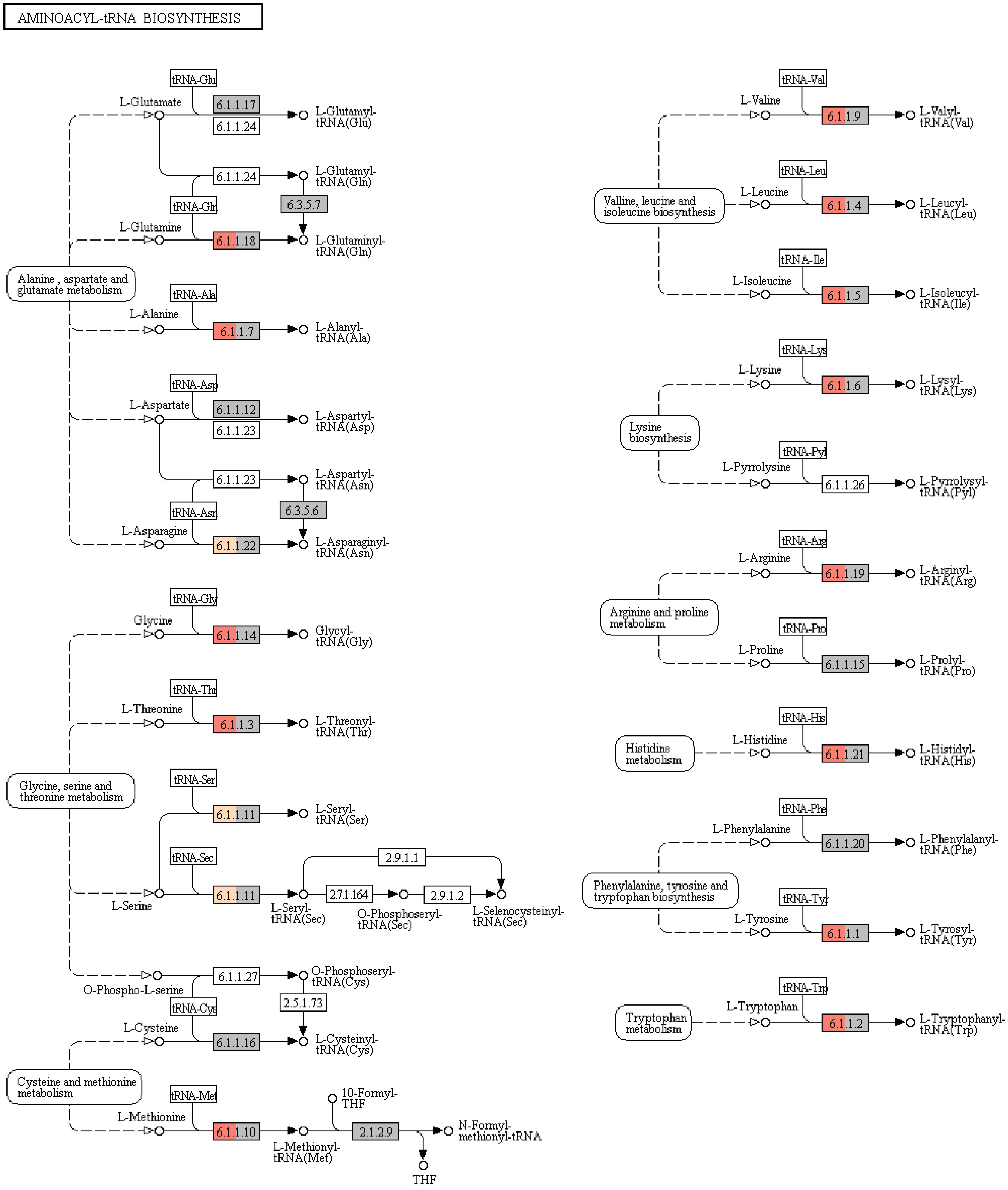

Data on KEGG graph
Rendered by Pathview
